# The *Drosophila ETV5* Homologue *Ets96B*: Molecular Link between Obesity and Bipolar Disorder

**DOI:** 10.1371/journal.pgen.1006104

**Published:** 2016-06-09

**Authors:** Michael J. Williams, Anica Klockars, Anders Eriksson, Sarah Voisin, Rohit Dnyansagar, Lyle Wiemerslage, Anna Kasagiannis, Mehwish Akram, Sania Kheder, Valerie Ambrosi, Emilie Hallqvist, Robert Fredriksson, Helgi B. Schiöth

**Affiliations:** Functional Pharmacology, Department of Neuroscience, Uppsala University, Uppsala, Sweden; Stanford University School of Medicine, UNITED STATES

## Abstract

Several reports suggest obesity and bipolar disorder (BD) share some physiological and behavioural similarities. For instance, obese individuals are more impulsive and have heightened reward responsiveness, phenotypes associated with BD, while bipolar patients become obese at a higher rate and earlier age than people without BD; however, the molecular mechanisms of such an association remain obscure. Here we demonstrate, using whole transcriptome analysis, that *Drosophila Ets96B*, homologue of obesity-linked gene *ETV5*, regulates cellular systems associated with obesity and BD. Consistent with a role in obesity and BD, loss of nervous system *Ets96B* during development increases triacylglyceride concentration, while inducing a heightened startle-response, as well as increasing hyperactivity and reducing sleep. Of notable interest, mouse *Etv5* and *Drosophila Ets96B* are expressed in dopaminergic-rich regions, and loss of *Ets96B* specifically in dopaminergic neurons recapitulates the metabolic and behavioural phenotypes. Moreover, our data indicate Ets96B inhibits dopaminergic-specific neuroprotective systems. Additionally, we reveal that multiple SNPs in human *ETV5* link to body mass index (BMI) and BD, providing further evidence for *ETV5* as an important and novel molecular intermediate between obesity and BD. We identify a novel molecular link between obesity and bipolar disorder. The *Drosophila ETV5* homologue *Ets96B* regulates the expression of cellular systems with links to obesity and behaviour, including the expression of a conserved endoplasmic reticulum molecular chaperone complex known to be neuroprotective. Finally, a connection between the obesity-linked gene *ETV5* and bipolar disorder emphasizes a functional relationship between obesity and BD at the molecular level.

## Author Summary

The World Health Organization suggests obesity is a major cause of poor health and is becoming the leading public health concern. Likewise, mood-based disorders, such as bipolar disorder, are one of the top ten causes of disability worldwide. There is evidence that obesity and bipolar disorder may be linked and that obesity may exacerbate bipolar disorder symptoms. For the first time, our work evidences a molecular-link between obesity and bipolar disorder. In humans the obesity-linked gene *ETV5* was also associated with bipolar disorder. Using the model organism *Drosophila melanogaster* (the fruit fly) we show that the *ETV5* homologue *Ets96B* regulates a series of genes known to be neuroprotective and inhibiting the expression of *Ets96* in dopaminergic neurons induces phenotypes linked to obesity and bipolar disorder, including increased lipid storage, increased anxiety and reduced sleep. Our work will help to further the understanding of how these to disorders may interact.

## Introduction

Clinical studies indicate there may be a link between obesity and bipolar disorder (BD). Obese individuals have greater impulsivity and reward responsiveness—phenotypes associated with BD [1, 2], while bipolar patients become obese and have cardiovascular disorders at a higher rate and earlier age than people without BD [3]. Although some studies link obesity and its sequelae to BD medication, a significant number of medication-naïve BD patients also display metabolic syndrome phenotypes [4, 5]. Moreover, BD patients have high rates of carbohydrate consumption and low rates of physical exercise [6, 7]. Hence, there seem to be several physiological and behavioural links between obesity and BD; however, the molecular mechanisms connecting these two diseases are not fully understood.

Ets variant 5 (*ETV5*, also known as ERM), an E-twenty-six (ETS) transcription factor belonging to the polyoma enhancer activator 3 (PEA3)-subfamily (*ETV1*, *ETV4* and *ETV5*), was initially linked to BMI in a GWAS study [8]. Experimental evidence in mice also links *Etv5* to the regulation of metabolic homeostasis; *Etv5* transcriptional expression is affected by ingestion of a high-fat/high-sugar diet, as well as food restriction [9, 10]. Moreover, *Etv5*^*-/-*^ mice exhibit decreased body weight compared to controls [9, 10]. In mouse brain, *Etv5* is expressed in the arcuate nucleus (ARC), ventro-medial hypothalamus (VMH), substantia nigra (SN), and the ventral tegmental area (VTA) [9]. Expression in the ARC and VTA implies involvement in reward-driven or energy-driven food intake [11]. Interestingly, these same regions regulate impulsivity [12]. Also, the SN is a key regulator of locomotion and disruptions in this region lead to hyperactivity [13].

The endoplasmic reticulum (ER) is involved in the biosynthesis, folding, modification and trafficking of proteins. Apart from this role there is emerging evidence that the ER is centrally involved in sensing metabolic changes within a cell and transmitting this information to the nucleus [14]. Preclinical and clinical studies in the last decade indicate that ER stress, including the unfolded protein response (UPR), has a significant impact on the pathogenesis of obesity [15, 16]. Additionally, recent studies suggest an involvement of UPR in the pathophysiology of BD [17, 18].

We turned to the genetically tractable model organism *Drosophila melanogaster* to understand more about the molecular mechanism of how the PEA3-family member *ETV5* may affect body weight and BD. In *Drosophila* the PEA3-family is represented by the homologue *Ets96B*. Almost nothing is known about *Ets96B*, but it is highly expressed in the central nervous system [19]. We knocked down *Ets96B* expression in the *Drosophila* nervous system and performed SOLiD sequencing of the entire transcriptome, discovering associations to gene networks whose human homologues are connected to body mass index (BMI) and bipolar disorder. Furthermore, loss of neuronal Ets96B induced lipid storage defects, hyperactivity, and a heightened startle-response phenotype. Interestingly, our data indicate that Ets96B works to suppress an ER molecular chaperone system shown to be neuroprotective in dopaminergic neurons [20, 21]. Finally, we reveal a human association of *ETV5* with bipolar disorder.

## Results

### Ets96B is a member of the PEA3 subfamily

**Fig 1:**
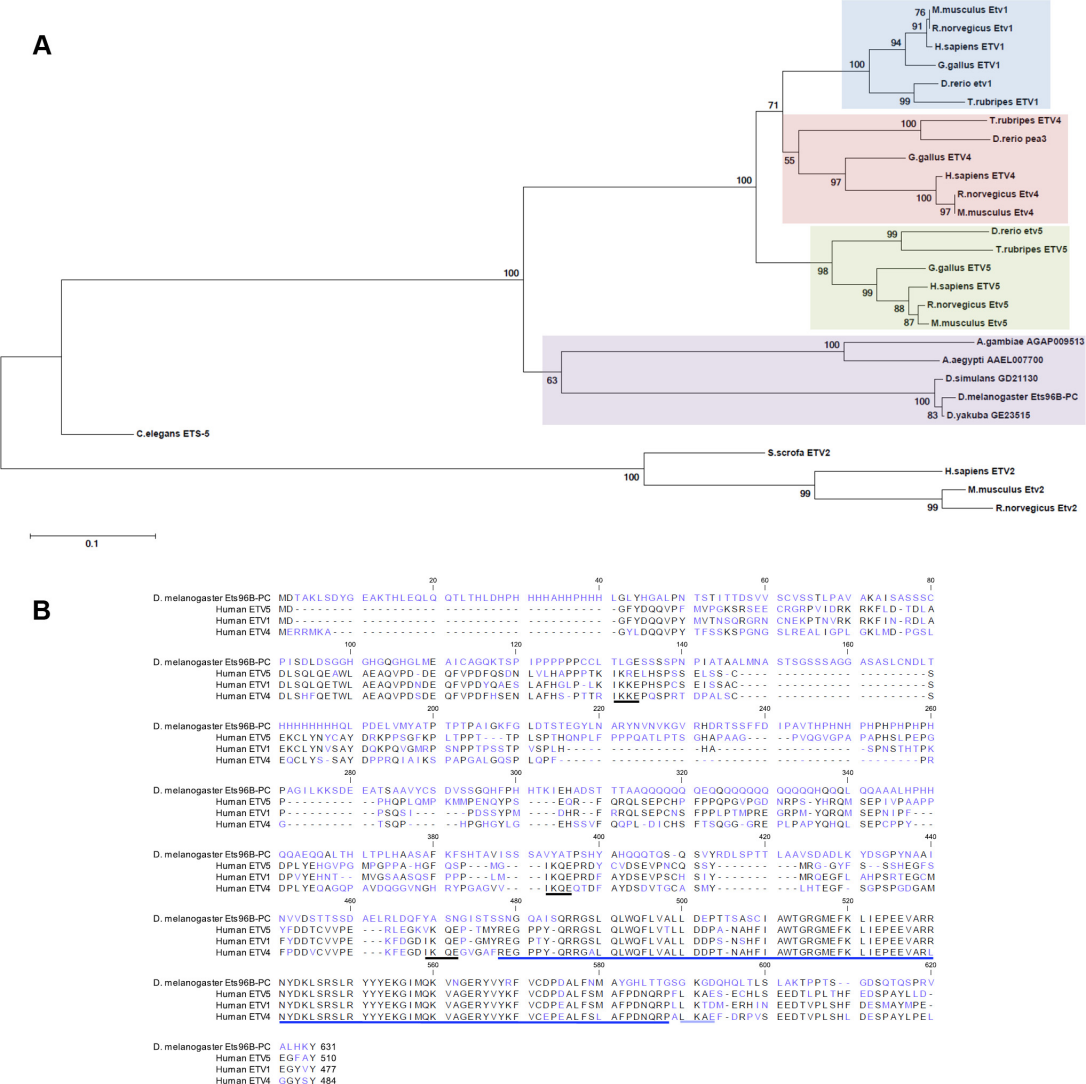
**Comparison of *Drosophila* Ets96B with mammalian PEA3-family members** (A) The evolutionary relationship was inferred using the Maximum-likelihood method. The optimal tree with the sum of branch length = 3.794 is shown. The percentage of replicate trees in which the associated taxa clustered together in the bootstrap test (5000 replicates) are shown next to the branches. The tree is drawn to scale, with branch lengths in the same units as those of the evolutionary distances used to infer the phylogenetic tree. (B) The protein sequences were aligned and edited using CLC Sequence Viewer 6. Colors correspond to amino acid conservation (black = conserved, blue = no conservation). The predicted ETS DNA binding domain is indicated by a dark blue underlining of amino acids near C-terminus. Human sumoylation sites are indicated by black underlining; predicted *Drosophila* sumoylation site is indicated by light blue underlining.

To begin our studies we confirmed that *Drosophila* Ets96B was homologous to human ETV5. Using the Maximum-likelihood method, the phylogenetic relationship between the *Drosophila* protein Ets96B and vertebrate PEA3-family proteins was determined (Fig 1A). All major vertebrate and invertebrate model organisms, where a PEA3-like protein existed that had an E-value of at least 1.0e-45 when compared to human ETV5, were included. The mammalian ETV2 subgroup was used as an outgroup. From the tree produced it was evident that invertebrates contained only one PEA3-family member, while the vertebrate family expanded to include three members: ETV1, ETV4, and ETV5. We also searched for PEA3-family homologues in the important model organisms *S*. *cerevisae* and *D*. *discoideum* by mining the NCBI’s protein database nr using protein PSI-BLAST with *Drosophila* Ets96B, as well as vertebrate ETV1, ETV4, and ETV5 protein sequences as queries, but no homologous proteins were discovered. From this data we conclude that *Drosophila melanogaster* Ets96B is basally related to the vertebrate PEA3-subfamily of ETS transcription factors.

A multiple sequence alignment was performed comparing *Drosophila* Ets96B with human PEA3-family proteins (Fig 1B). This alignment demonstrated that the ETS domain at the C-terminus, found in all ETS-family proteins, was well conserved (Fig 1B, dark blue underlining). Although some conservation was observed in the N-terminus, the PEA3 domain, present in all mammalian PEA3 family members, was not conserved. In regards to regulatory conservation, mammalian ETV5 is sumoylated on multiple sites, necessary for the inhibition of ETV5 activity [22]. These sumoylation sites were conserved in all human PEA3-family members (Fig 1B, black underlining). Using the Abgent sumoplot (http://www.abgent.com/sumoplot), as well as GPS-Sumo [23], we determined that most of the mammalian sumoylation sites were not conserved in Ets96B. *Drosophila* Ets96B had a predicted site at lysine 429 (K429) with the sequence LKQD, and a site (K590) possibly conserved with human PEA3-family members, having the sequence GKGD (Fig 1B, light blue underlining). Whether or not these Ets96B sites are actually sumoylated is not known.

### Differential gene expression in Ets96B knockdowns

**Fig 2:**
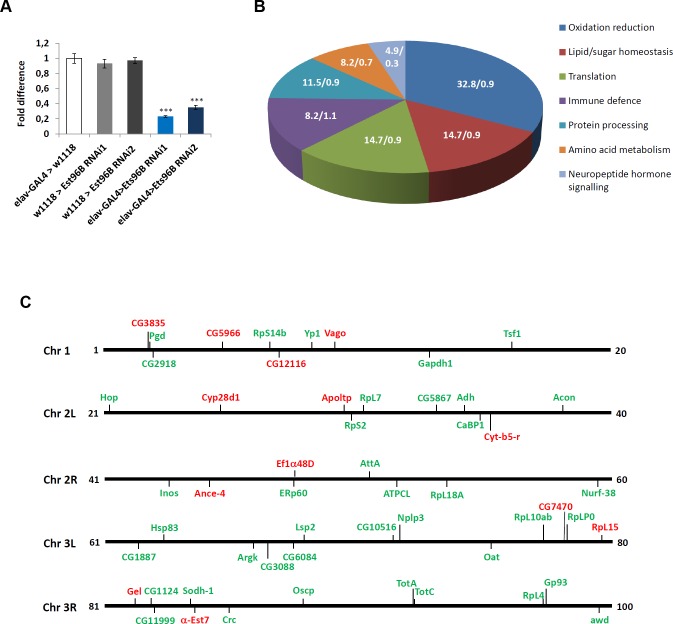
***Ets96B* regulates genes involved in oxidative phosphorylation and redox reactions** (A) Relative expression level of *Ets96B* in 5–7 day old control males or males where *Ets96B* was knocked down in the entire nervous system throughout development. This assay was repeated at least 7 times. (n = 25 males per treatment; ** P<0.005 compared with controls, one-way ANOVA with Tukey’s post hoc test for multiple comparisons). Error bars = SEM. (B) Pie chart showing KEGG classification of gene groups (percentage of total Ets96B up or down regulated genes /percentage of all genes in the genome belonging to a particular category). (C) *Drosophila* genome depicting the location of genes up (green) or down (red) regulated in Ets96B SOLiD sequencing of the entire *Drosophila* transcriptome.

*Ets96B* is highly expressed in the nervous system [19], therefore we knocked it down in all neuronal cells throughout development, using the pan-neuronal *elav-GAL4* driver [24] to express *UAS-Ets96B*^*RNAi1*^ (*Ets96B*^*RNAi1*^) [25], and then performed SOLiD sequence analysis of the entire transcriptome. By mapping the *Drosophila* transcriptome to the reference genome obtained from flybase (build dmel_r5.47_FB2012_05), 15147 transcripts were identified, including expressed genes (mRNA), miRNA, snRNA, snoRNA and tRNA (S1 Dataset). The *Drosophila* gene *Ets96B* encodes two transcript variants (FBtr0084696 and FBtr0084697); however, the adult samples we sequenced expressed only FBtr0084696 (Ets96B-RC), which encodes the Ets96B-PC protein isoform. Ets96B-PC is identical to Est96B-PB except for the extreme N-terminus, where Ets96B-PB had a nine amino acid insert. In this experiment, 166 genes were differentially expressed, taking into consideration the 'false discovery rate' and correcting for it using Benjamini-Hochberg correction (S1 Dataset). We observed a complete lack of expression of *Ets96B* in the experimental sample (S1 Dataset). To confirm the SOLiD result we performed quantitative RT-PCR (qPCR) to measure the level of *Ets96B* transcript. Compared with controls, *Ets96B* transcript levels were significantly lower in *elav-GAL4;UAS-Ets96B*^*RNAi1*^ males (P < 0.005) (Fig 2A). At this time we also checked *Ets96B* expression levels using a second *UAS-Ets96B* RNAi line (*Ets96B*^*RNAi2*^) [25], transcript levels were also significantly lower in the heads of *elav-GAL4;UAS-ETS96B*^*RNAi2*^ males (P < 0.005) (Fig 2A).

After performing pair-wise comparisons between all sequenced strains, 61 differentially expressed genes remained significant (Table 1). Interestingly, most of these genes were up-regulated when *Ets96B* was knocked down in the CNS, with only 21% being down-regulated. Using DAVID and KEGG, we categorized the differentially expressed genes by function [26, 27]. The largest group was involved in oxidative phosphorylation and redox reactions, with 32.8 percent of the genes falling into this category (Fig 2B). The next largest groups were a tie between genes involved in lipid and sugar homeostasis, as well as those involved in translation, each contained 14.7% of the genes (Fig 2B). Next, we mapped the differentially expressed genes to identify if they were enriched in any particular chromosomal region (Fig 2C). From this map it was evident that genes affected by loss of *Ets96B* were distributed throughout the genome.

**Table 1:** Solid sequencing data for up and down regulated genes in *Ets96B*^*RNAi*^ versus all controls

Biological Process	ID	Gene Name	Ets96B^RNAi^	Expressed in CNS	Human homologue
			log2 (fold change)	according to FlyAtlas	
**Lipid/sugar homeostasis**					
	FBgn0029831	CG5966	-2,91	Yes	*PNLIP*
	FBgn0015575	α-Est7	-0,99	Yes	
	FBgn0032136	Apoltp	-0,65	Yes	
	FBgn0037312	CG11999	3,36	Yes	*SDF2*
	FBgn0036549	CG10516	2,05	Yes	*SPSB3*
	FBgn0004654	Pgd	2,03	Yes	*PGD*
	FBgn0025885	Inos	1,29	Yes	*ISYNA1*
	FBgn0004045	Yp1	1,10	Yes	
	FBgn0001092	Gapdh2	0,75	Yes	*GAPDH*
**Translation**					
	FBgn0028697	RpL15	-0,70	Yes	*RPL15*
	FBgn0000556	Ef1α48D	-0,59	Yes	*EEF1A1*
	FBgn0010409	RpL18A	1,28	Yes	*RPL18A*
	FBgn0004404	RpS14b	1,13	Yes	*RPS14*
	FBgn0004867	RpS2	0,76	Yes	*RPS2*
	FBgn0003279	RpL4	0,66	Yes	*RPL4*
	FBgn0036213	RpL10Ab	0,64	Yes	*RPL10A*
	FBgn0005593	RpL7	0,63	Yes	*RPL7*
	FBgn0000100	RpLP0	0,58	Yes	*RPLP0*
**Amino acid metabolism**					
	FBgn0037146	CG7470	-1,51	Yes	*ALDH18A1*
	FBgn0002565	Lsp2	3,91	Yes	
	FBgn0022774	Oat	2,26	Yes	*OAT*
	FBgn0000150	awd	1,36	Yes	*NME1-NME2*
	FBgn0000116	Argk	0,76	Yes	*CKB*
**Oxidation reduction**					
	FBgn0023507	CG3835	-1,53	Yes	*D2HGDH*
	FBgn0030041	CG12116	-1,22	Yes	
	FBgn0031689	Cyp28d1	-1,11	Yes	
	FBgn0000406	Cyt-b5-r	-1,06	Yes	
	FBgn0025678	CaBP1	3,24	Yes	*CABP1*
	FBgn0033663	ERp60	2,57	Yes	*PDIA3*
	FBgn0013685	mt:ND6	2,13	Yes	*ND6*
	FBgn0013684	mt:ND5	1,57	Yes	*ND5*
	FBgn0013680	mt:ND2	1,38	Yes	*ND2*
	FBgn0013675	mt:CoII	1,30	Yes	*COX2*
	FBgn0086254	CG6084	1,29	Yes	*AKR1B15*
	FBgn0013678	mt:Cyt-b	1,26	Yes	*CYTB*
	FBgn0016691	Oscp	1,16	Yes	*ATP5O*
	FBgn0013672	mt:ATPase6	1,11	Yes	*ATP6*
	FBgn0020236	ATPCL	1,00	Yes	*ACLY*
	FBgn0010100	Acon	0,98	Yes	*ACO2*
	FBgn0013674	mt:CoI	0,96	Yes	*COX1*
	FBgn0024289	Sodh-1	0,96	Yes	*SORD*
	FBgn0000055	Adh	0,76	Yes	*ADH1*
	FBgn0013676	mt:CoIII	0,74	Yes	*COX3*
**Protein processing**					
	FBgn0033366	Ance-4	-1,31	Yes	
	FBgn0005585	Crc	2,75	Yes	*CALR*
	FBgn0001233	Hsp83	2,19	Yes	*HSP90AB1*
	FBgn0036015	CG3088	1,99	Yes	
	FBgn0039562	Gp93	1,27	Yes	*HSP90B1*
	FBgn0024352	Hop	2,06	Yes	*STIP1*
	FBgn0023529	CG2918	1,37	Yes	*HYOU1*
**Immune defense**					
	FBgn0030262	Vago	-1,78	Yes	
	FBgn0010225	Gel	-0,58	Yes	
	FBgn0044812	TotC	3,71	Yes	
	FBgn0012042	AttA	2,75	Yes	
	FBgn0028396	TotA	1,43	Yes	
	FBgn0035290	CG1887	1,16	No	
	FBgn0022355	Tsf1	0,97	Yes	
	FBgn0016687	Nurf-38	0,93	Yes	*PPA2*
**Neuropeptide hormone signaling**					
	FBgn0037290	CG1124	1,35	Yes	
	FBgn0027586	CG5867	0,96	Yes	
	FBgn0042201	Nplp3	0,81	Yes	

### Ets96B inhibits the expression of a conserved molecular chaperone complex

**Fig 3:**
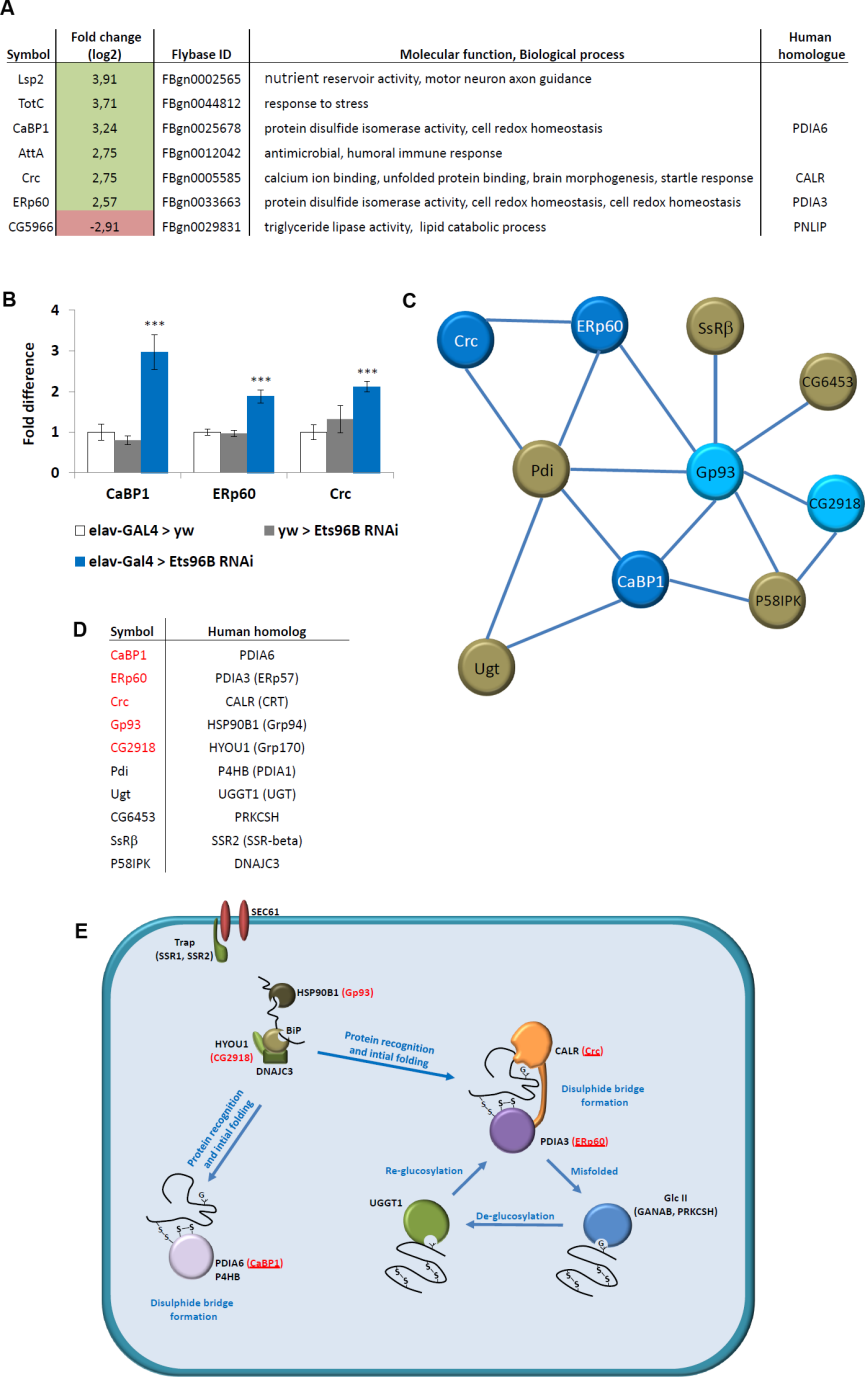
*Ets96B* regulates a conserved molecular chaperone complex. (A) SOLiD data for *elav-GAL4>Ets96B*^*RNAi*^ versus controls (*w*, *elav-GAL4*>*w*^*1118*^ and *w*^*1118*^> *Ets96B*^*RNAi*^) 5–7 day old male heads. Only genes regulated more than ±log2 2.5-fold change are included. (B) Relative expression levels of *CaBP1*, *ERp60* and *Crc* in 5–7 day old control males or males where *Ets96B* was knocked down in the entire nervous system throughout development. This assay was repeated at least 7 times. Error bars indicate SEM. (n = 25 males per treatment; *** P<0.005 compared with *elav-GAL4* controls, one-way ANOVA with Tukey’s post hoc test for multiple comparisons). (C) Biogrid and String were used to find all genes that interact with the molecular chaperone genes recovered in the *Ets96B* knockdown males. Genes in red were recovered in the *Ets96B* SOLiD sequencing. (D) Data showing human homologues of Drosophila genes recovered in Biogrid and String analysis. Genes in red were recovered in the *Ets96B* SOLiD sequencing. (E) Diagram depicting molecules involved in endoplasmic reticulum protein folding. These include molecular chaperones that function to prevent aggregation (Bip, HSP90B1, DNAJC3, HYOU1 and CALR), protein disulfide isomerases that catalyze disulfide bond formation, isomerization and reduction (PDIA3, PDIA3 and P4HB), and proteins involved in de- or re-glycosylation of improperly folded glycoproteins (PRKCSH and UGGT1). *Drosophila* homologues that were up-regulated when *Ets96B* was knocked down are in red, and those that were up-regulated more than 2.5-fold are underlined.

Using a ±log2 change in expression of 2.5 as a cut-off, we investigated the genes most highly affected when *Ets96B* was knocked down in the entire nervous system throughout development (Fig 3A). We utilized STRING and BioGrid [28, 29] to find interacting proteins for these top hits, however, it was immediately evident that three of them interacted: *Calcium-binding protein 1* (*CaBP1*), *Calreticulin* (*Crc*), and *ERp60*.

To confirm the SOLiD sequencing results we picked *CaBP1*, *Crc* and *ERp60* and performed qPCR analysis (Fig 3B). The level of expression in *elav-GAL4* heterozygous controls was set as 100%, represented as 1 on the graph (Fig 3B). Compared to either *elav-GAL4*^*+/-*^ or *Ets96B*^*RNAi1+/-*^ controls, *Ets96B*^*RNAi1*^ knockdown flies had a significant increase in *CaBP1* (P < 0.005), *ERp60* (P < 0.005) and *Crc* (P < 0.005) expression (Fig 3B). This was similar to results obtained by SOLiD sequencing (Fig 3A).

It was reported that overexpression of an RNAi can lead to saturation of the RNA-induced silencing complex (RISC), leading to neuronal cell death or stress [30, 31]. Therefore, we performed a control experiment overexpressing GFP under the control of *elav-GAL4* and then used two different GFP RNAi lines to inhibit GFP expression (Figs 1A and S3). We then measured the level of expression of *CaBP1*, *ERp60* and *Crc*; no differences in the expression levels of *CaBP1*, *ERp60* and *Crc* were observed between controls and GFP knockdown males (Figs 1B and S3).

When looking at interacting genes, we noticed that *Crc* networked in both STRING and BioGRID with another hit from the SOLiD sequence data, *Glycoprotein 93* (*Gp93*, human *HSP90B1*), which in turn interacted with yet another hit, *CG2918* (human *HYOU1*) (Table 1). Utilizing these genes, and all the genes with which they were predicted to interact, we employed the STRING program to make a network (Fig 3C). From this it was evident that the genes formed a network, with *Gp93*, *Protein disulfide isomerise* (*Pdi*), and *CaBP1* identified as hubs (Fig 3C).

All of the genes used to make the interaction map had human homologues (Fig 3D). These proteins locate to the ER and are involved in the regulation of protein folding (Fig 3E). Of note, all homologous genes in this pathway that were highly overexpressed when *Ets96B* was knocked down in the CNS, are involved in disulphide bridge formation (*Crc*, *ERp60* and *CaBP1*) (Fig 3E, proteins marked in red and underlined).

### Ets96B affects starvation resistance

Given that knocking down *Ets96B* affected genes involved in energy homeostasis (mitochondrial electron transport chain, Table 1) and protein production (Fig 3E), as well as inhibited the expression of *CG5966* (*Drosophila* homologue of human *pancreatic lipase*, *PNLIP*), we wanted to understand if loss of *Ets96B* had any effect on metabolism. Furthermore, to determine if *Ets96B* had a developmental phenotype, both the pan-neuronal *elav-GAL4* driver and the *Ets96B-GAL4* driver [32] were employed. *Ets96B* is located on the right arm of the third chromosome at 96A22 in the forward orientation and encodes for two isoforms (S1 Fig). Recently, a 832 bp region from the third intron of Ets96B-RB, or fourth intron of Ets96B-RC, was cloned and used to make a GAL4 transgenic line (*GMR36H05-GAL4*, which we refer to as *Ets96B-GAL4*) [32] (S1 Fig).

**Fig 4:**
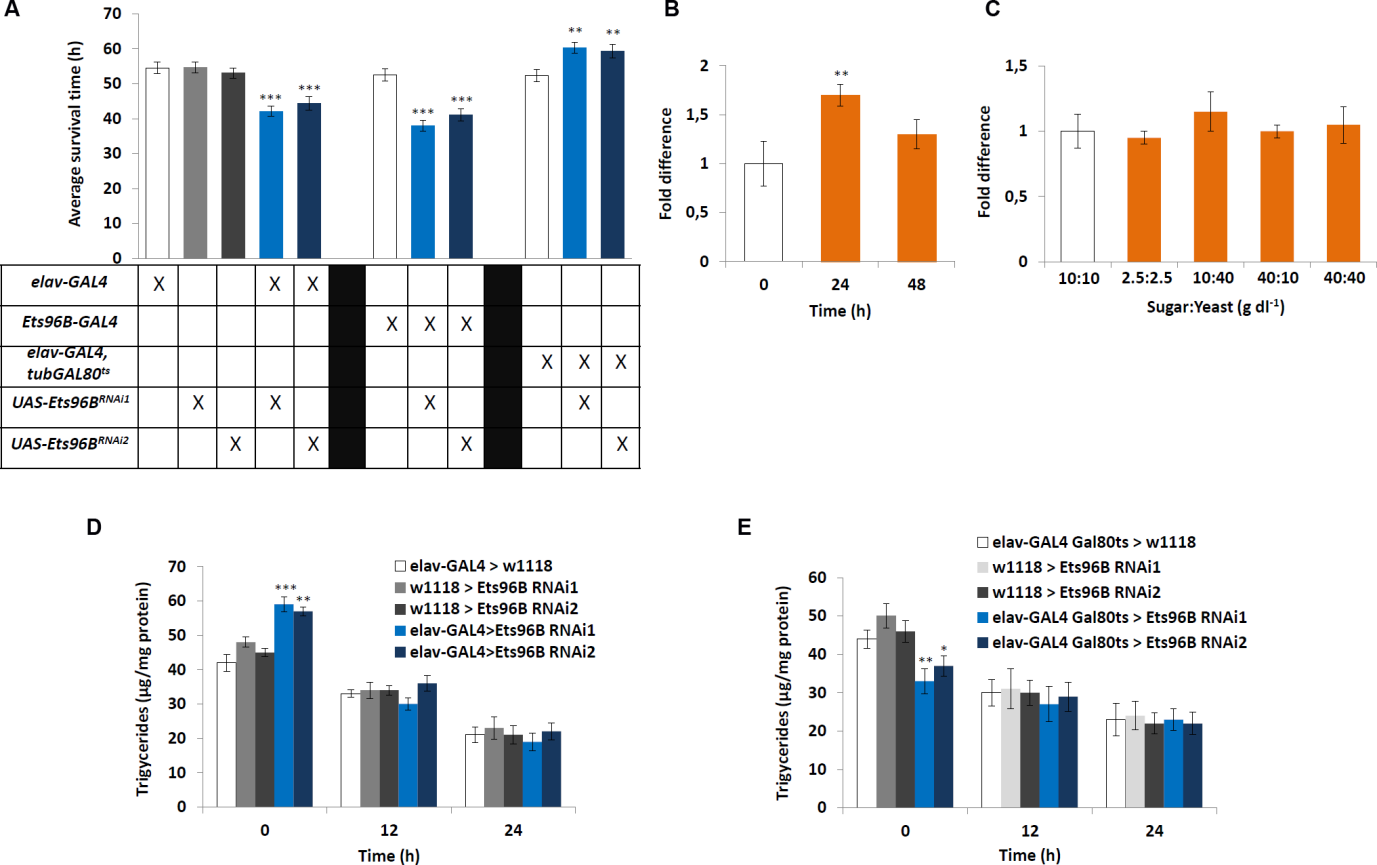
*Ets96B* regulates starvation resistance. (A) We tested the effect of *Ets96B*-knockdown in flies using the starvation survival assay. 5–7 day old control and *Ets96B* knockdown males (GAL4 driver crossed to either *Ets96B*^*RNAi1*^ or *Ets96B*^*RNAi2*^) were placed in a vial containing 1% agarose and maintained at 25°C. DAMS was used to monitor activity. (n = 30–60 flies per genotype, one-way ANOVA with Tukey’s post hoc test for multiple comparisons) (B) To examine how starvation affects the expression level of *Ets96B*, RNA was extracted from normal fed flies, as well as after 24 and 48 h of starvation. (C) To examine how nutritional state affects the expression levels of *Ets96B*, RNA was extracted under different nutritional states. Flies fed ad lib were set as 100%, represented by 1 on the graphs (B, C: n = 10 replicates, one-way ANOVA with Tukey’s post hoc test for multiple comparisons) (D, E) Triglyceride levels were determined in male flies at 0, 12 and 24 hours of starvation. The (D) *elav-Gal4* driver was used to express Ets96B RNAi (*Ets96B*^*RNAi1*^ or *Ets96B*^*RNAi2*^*)* throughout development (UAS-*Ets96B*^*RNAi1*^ and UAS-*Ets96B*^*RNAi2*^) and (E) the *elav-GAL4*,*tub-GAL80*^*ts*^ driver was used to knockdown *Ets96B* (*Ets96B*^*RNAi1*^ or *Ets96B*^*RNAi2*^*)* in adult males. (D, E: n = 30 males per treatment, assay was repeated at least 10 times for each genotype, one-way ANOVA with Tukey’s post hoc test for multiple comparisons). In all graphs significance levels are indicated: *, *P*<0.05; **, *P*<0.01; ***, *P* <0.005. Error bars = SEM.

To begin, we performed a starvation assay utilizing the Drosophila Activity Monitoring System (DAMS), as it is able to uncover broad defects in metabolism [33]. Knocking down *Ets96B* in the entire CNS during development using either *Ets96B*^*RNAi1*^ or *Ets96B*^*RNAi2*^ made flies more susceptible to starvation (P < 0.005) (Fig 4A). A similar phenotype was observed when *Ets96B* was knocked down throughout development using the *Ets96B-GAL4* driver (P < 0.005) (Fig 4A). To clarify if this was indeed a developmental phenotype, we used the *elav-Gal4*,*tubGal80*^*ts*^ driver [34] to knockdown *Ets96B* specifically in adults using either *Ets96B*^*RNAi1*^ or *Ets96B*^*RNAi2*^. Remarkably, this induced the opposite phenotype, where males were significantly more resistant to starvation than controls (P < 0.01) (Fig 4A).

To establish if *Ets96B* transcript levels in the brain were regulated by starvation, we performed qPCR analysis on heads from adult males starved for either 24h or 48h. Starving males for 24h significantly increased *Ets96B* expression (P < 0.01) (Fig 4B). Additionally, we determined if macronutrient content influenced *Ets96B* expression. Transcript levels of males fed a control diet (10 g/dl:10 g/dl sucrose:brewer’s yeast) were set as 100%, represented as 1 on the graph (Fig 4C). However, none of the diets had a significant effect on *Ets96B* expression.

### Ets96B affects lipid storage

Given that starvation resistance was affected in *Ets96B* knockdown males, to understand if *Ets96B* regulates normal feeding behaviour a CAFE assay was performed to measure how much food flies fed *ad libitum* consumed during a 24h period [35]. There was no significant difference in the amount of food consumed when *Ets96B* was knocked down in the nervous system by expressing *Ets96B*^*RNAi1*^ throughout development (S2 Fig). We also performed the CAFE assay using the *elav-Gal4*,*tubGAL80*^*ts*^ driver to knockdown *Ets96B* specifically in adults. Again no significant difference was observed between *Ets96B* knockdown flies or controls (S2 Fig). Perhaps these are not surprising results, given that our SOLiD data revealed no significant change in the expression of any gene known to directly influence feeding behaviour (Table 1 and S1 Dataset).

Next, we determined the stored lipid content before and during starvation. For this we measured triacylglyceride (TAG) levels in normal fed flies, as well as during starvation. Interestingly, the lipid content of *elav-GAL4*;*UAS-Ets96B*^*RNAi1*^ or *elav-GAL4*;*UAS-Ets96B*^*RNAi2*^ knockdown males was significantly higher than either control (P < 0.005) (Fig 4D). After 12h and 24h of starvation all strains had lower total TAG levels and there was no significant difference between strains (Fig 4D). This suggests that during the first 12h of starvation, *Ets96B*^*RNAi*^ knockdown males mobilized more of their stored lipids than control flies.

Next, using the *elav-Gal4*,*tubGal80*^*ts*^ driver, we knocked down *Ets96B* specifically in adults. Similar to the starvation assay, this induced the opposite phenotype, where the TAG content of *Ets96B*^*RNAi1*^ or *Ets96B*^*RNAi2*^ knockdown males was significantly lower than either control (*Ets96B*^*RNAi1*^ P < 0.01, *Ets96B*^*RNAi2*^ P < 0.05) (Fig 4E). As before, after 12h and 24h of starvation all strains had lower lipid contents and there was no significant difference between strains (Fig 4E). Contrary to knocking down *Ets96B* throughout development, when *Ets96B* was knocked down specifically in the adult nervous system, males mobilized significantly less of their lipid stores during the first 12h of starvation.

Even though knocking down a random gene, such as EGFP, in the nervous system had no effect on the Ets96B-regulated molecular chaperones, *CaBP1*, *ERp60* or *Crc*, we still wanted to confirm that activating the RNAi system in the CNS didn’t induce unspecific phenotypes. Therefore, we measured triacylglyceride (TAG) levels in normal fed flies, as well as during starvation; of controls (*elav-GAL4;UAS-GFP*) and GFP knockdown males, no differences in TAG levels were observed (Figs 3C and S3).

### Ets96B regulates startle-response and sleep

**Fig 5:**
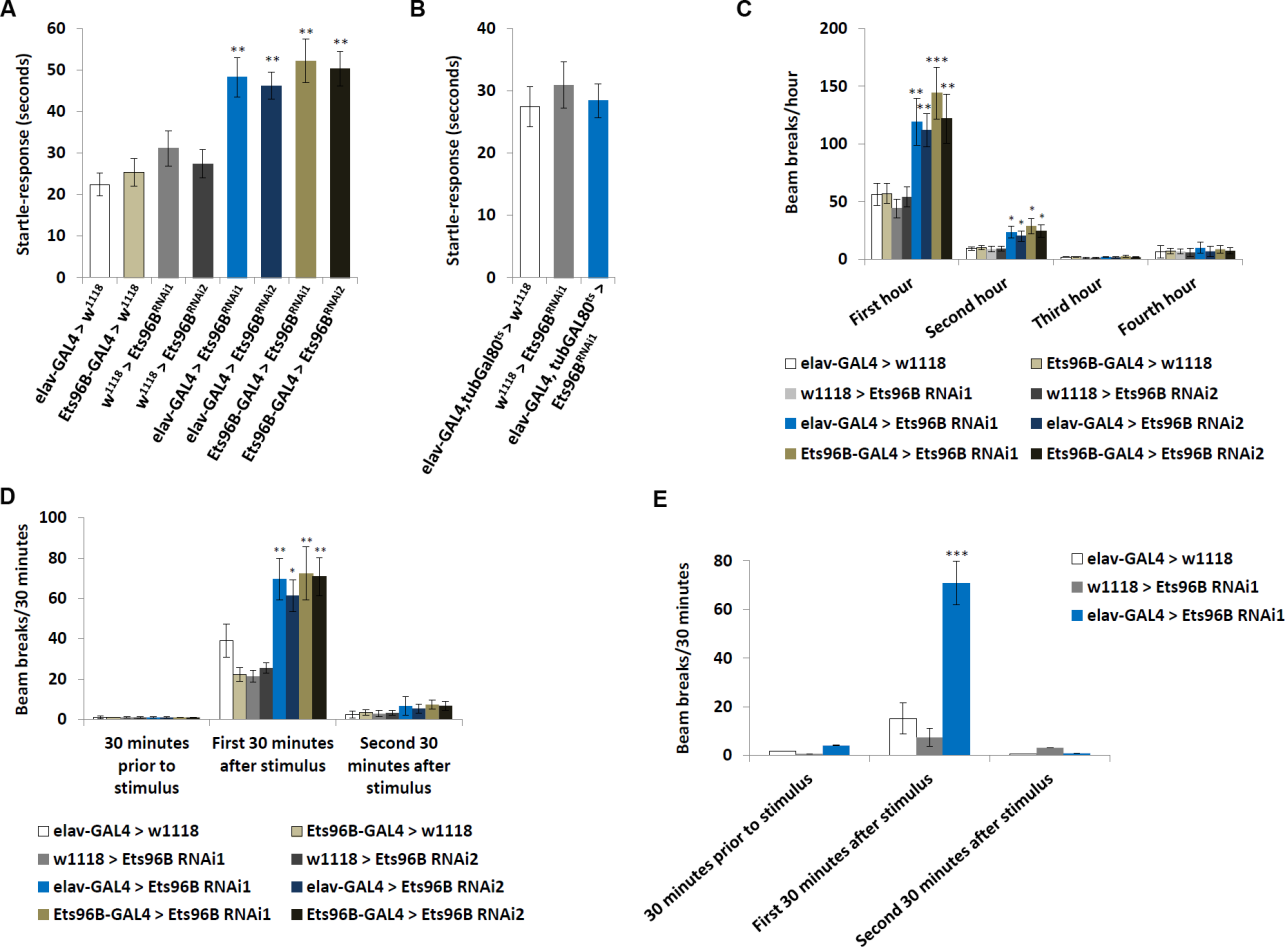
*Ets96B* knockdown during development induces a heightened startle-response. (A, B) Startle-response test demonstrating *Ets96B* knockdown males have a hyperactive startle-response. (A) *Ets96B* knocked down throughout development using UAS-*Ets96B*^*RNAi1*^ or UAS-*Ets96B*^*RNAi2*^ crossed to the pan-neuronal driver *elav-GAL4* (B) *Ets96B* knocked down only in adults using UAS-*Ets96B*^*RNAi1*^ crossed to the pan-neuronal driver and temperature sensitive allele of the GAL4 inhibitor GAL80 *elav-GAL4*, *tub-Gal80*^*ts*^. (A, B: n = 50 males per strain; ** P<0.01 compared with controls, one-way ANOVA with Tukey’s post hoc test for multiple comparisons). (C) The DAMS system was used to monitor locomotion for the first four hours flies were placed in the system. (D) The DAMS system was used to monitor locomotion prior to and after light stimulation. (E) The DAMS system was used to monitor locomotion prior to and after sound stimulation (65–70 dB). Only *Ets96B*^*RNAi1*^ was used for this assay. (C-E: n = 30–60 males per strain; * P < 0.05, ** P<0.01, *** P < 0.005 compared with controls, one-way ANOVA with Tukey’s post hoc test for multiple comparisons) In all graphs error bars = SEM.

One of the molecular chaperones upregulated when *Ets96B* was knocked down in the nervous system, *Crc*, is necessary for startle-induced locomotion. Yamamoto et al. [36] reported that *Crc* mutants lack a startle-response. Therefore, we hypothesized that *Ets96B* knockdown males, where *Crc* is overexpressed (see Fig 3A and 3B), would have the opposite phenotype and be excessively responsive to being startled. Using the same assay [36], we found that knocking down *Ets96B* in the nervous system throughout development induced a heightened startle-response (P < 0.01) (Fig 5A). When *Ets96B* was knocked down specifically in adults the startle-response was the same as control males (P = 0.57) (Fig 5B).

Ets96B was further assessed for behavioural defects using a DAMS movement based assay. During the first hour in the DAMS knocking down *Est96B* (*Ets96B*^*RNAi1*^ or *Ets96B*^*RNAi2*^), using either *elav-GAL4* or *Ets96B-GAL4*, had significantly more movement than controls (P < 0.01, except *Ets96B-GAL4;UAS-Ets96B*^*RNAi1*^ P < 0.005) (Fig 5C) This hyperactivity continued during the second hour in the DAMS (P < 0.05) (Fig 5C). The activity level of all genotypes was similar throughout the last two hours monitored. Furthermore, during the first 30 minutes after light shock, both *elav-GAL4;UAS-Ets96B*^*RNAi*^ and *Ets96B-GAL4;UAS-Ets96B*^*RNAi*^ knockdown males, using either *Ets96B*^*RNAi1*^ or *Ets96B*^*RNAi2*^, had significantly more movement than either control (P < 0.01, except *elav-GAL4;UAS-Ets96B*^*RNAi1*^ P < 0.05), but not 30 minutes prior to or 30–60 minutes after light shock (Fig 5D). This indicates that knocking down *Ets96B* in the nervous system during development increased exploration/anxiety when the setting was novel, as well as after a light-shock stimulus.

Next, to check that the DAMS measured response was not specific for light-shock stimulus, we repeated the assay using a loud white noise (65-70dB) to induce stress. Similar to what was observed with light-stimulus, during the first 30 minutes after noise shock, *elav-GAL4;UAS-Ets96B*^*RNAi1*^ knockdown males had significantly more movement than either control (P < 0.005), but not 30 minutes prior to or 30–60 minutes after light shock (Fig 5E)

Finally, to check for any general effects on the neurons due to overexpression of an RNAi, we again expressed GFP under the control of *elav-GAL4* and used two different GFP RNAi lines to inhibit GFP expression. We then tested these males in the DAMS for any effect on the startle-response. Similar to what was observed in the TAG assay, general overexpression of an RNAi in the entire nervous system had no effect on the startle-response (Figs 3D and S3).

**Fig 6:**
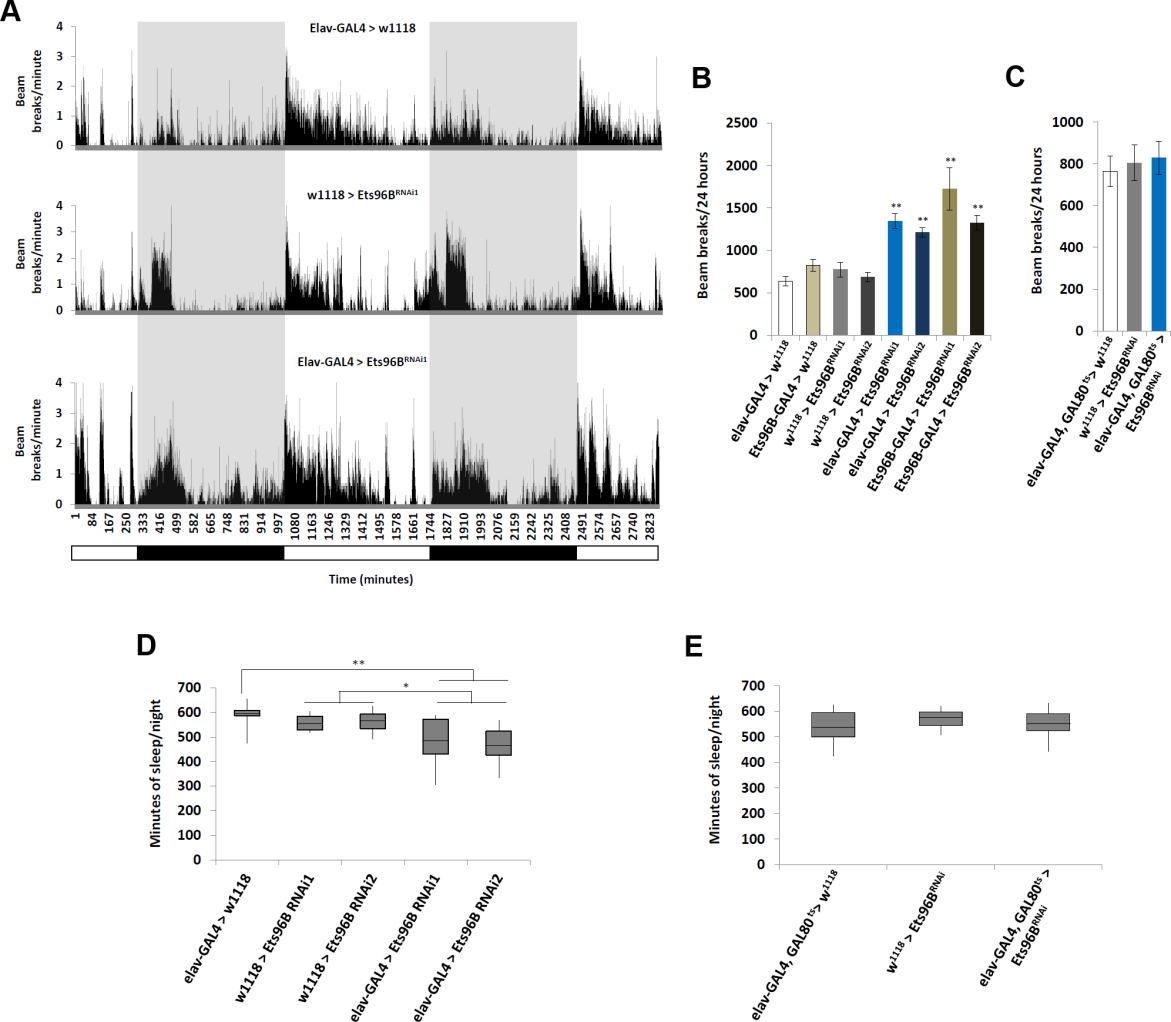
*Ets96B* regulates activity and sleep/wake behaviour. The DAMS system was used to monitor locomotion and sleep/wake behaviour over a 48 hour period. (A) Bar diagram indicating general activity over a 48 hour period of adult flies maintained on a 12:12 hour light:dark cycle. White bar indicates lights-on and dark bar and gray highlight indicate lights-off. Representative result for one run using *Ets96B*^*RNAi1*^ is shown (B) Total number a beam breaks over a 24 hour period in control flies or flies were Ets96B was knocked down (*Ets96B*^*RNAi1*^ or *Ets96B*^*RNAi2*^) throughout development either in the entire nervous system (*elav-GAL4*) or specifically in Ets96B neurons (*Ets96B-GAL4*). (C) Total number a beam breaks over a 24 hour period in control flies or flies were Ets96B was knocked down (*Ets96B*^*RNAi1*^) specifically in adult neurons (*elav-GAL4;tubGAL80*^*ts*^). (D) Total minutes flies slept per night in control flies or flies were Ets96B was knocked down (*Ets96B*^*RNAi1*^ or *Ets96B*^*RNAi2*^) throughout development, either in the entire nervous system (*elav-GAL4*) or specifically in Ets96B neurons (*Ets96B-GAL4*). (E) Total minutes flies slept per night in control flies or flies were Ets96B was knocked down (*Ets96B*^*RNAi1*^) specifically in adult neurons (*elav-GAL4;tubGAL80*^*ts*^). (B-E, n = 30–60 males per strain; * P < 0.05, ** P<0.01 compared with controls, one-way ANOVA with Tukey’s post hoc test for multiple comparisons) In all graphs error bars = SEM.

In addition, we employed the DAMS to determine if there was an effect on general activity and sleep/wake behaviour (Fig 6A). To measure locomotor activity, male flies were placed in the DAMS and activity was quantified every minute for a 24h period. When *Ets96B* was knocked down using either the *elav-GAL4* (P < 0.01) or *Ets96B-GAL4* (P < 0.01) driver, males were significantly more active (Fig 6B). When *Ets96B* was knocked down specifically in adults, activity was similar to controls (P = 0.58 compared to *elav-Gal4*,*tubGal80*^*ts*^ heterozygous control) (Fig 6C). We also observed that *elav-GAL4 > Ets96B*^*RNAi*^ knockdown flies (*Ets96B*^*RNAi1*^ or *Ets96B*^*RNAi2*^) slept significantly less during the night than controls (Fig 6A and 6D). When *Ets96B* was specifically knocked down in adults there was no difference in sleep/wake behaviour between knockdown males and controls (Fig 6E, only *Ets96B*^*RNAi1*^ used for this experiment).

### *Drosophila* Ets96B and mouse Etv5 are expressed in dopaminergic neurons

**Fig 7:**
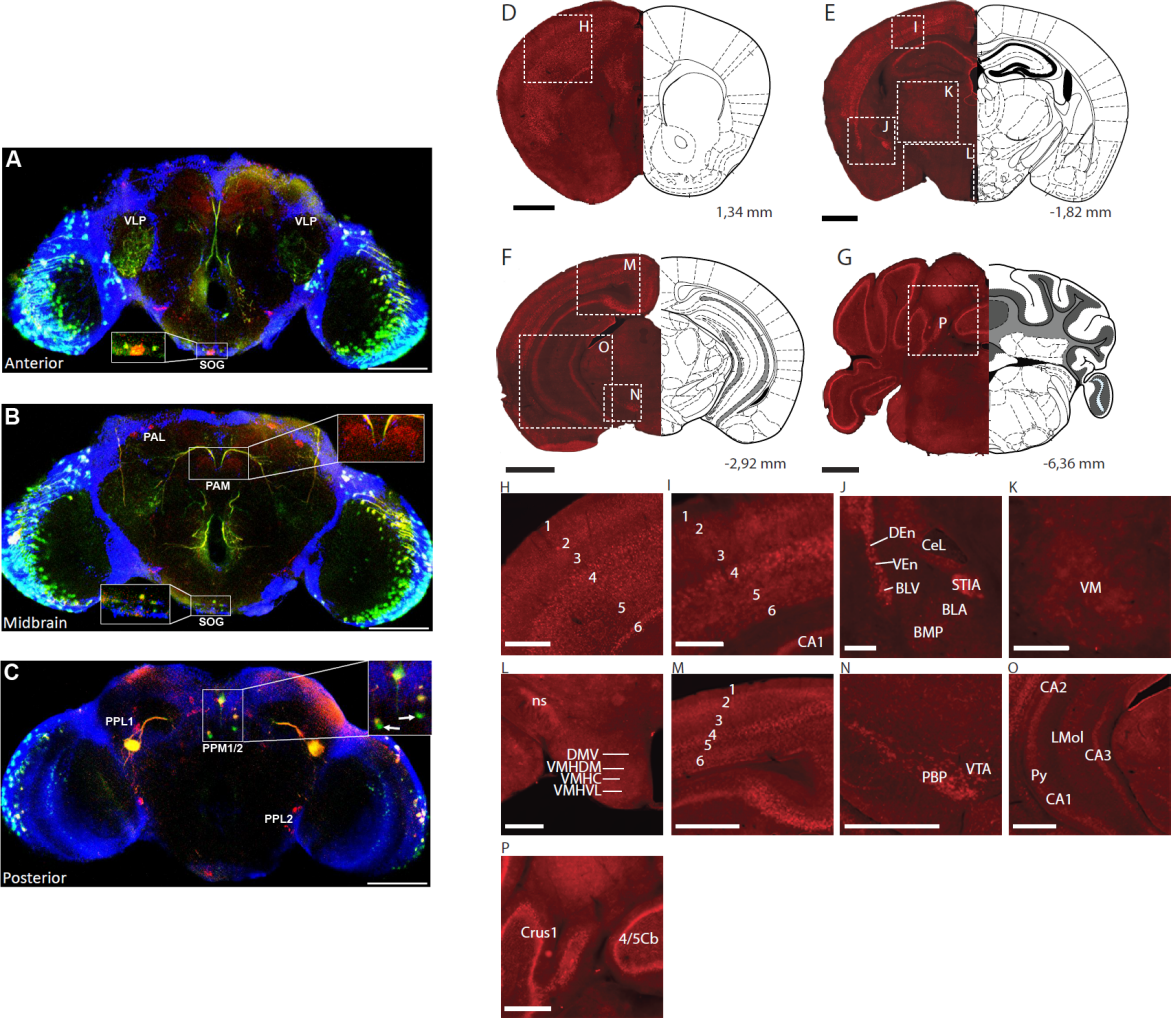
*Ets96B* and *Etv5* expressed on dopaminergic neurons. (A-C) *Ets96B-GAL4* was crossed to *UAS-GFP* to examine *Ets96B* expression in adult male brains. Adult male brains were then stained for GFP and Tyrosine hydroxylase (TH) expression. (A) Anterior section showing co-expression of GFP and TH in the eye, the ventrolateral prototcerebrum (VLP and the suboesophageal ganglion (SOG, see inset). (B) Midbrain, no co-expression was observed in the dopaminergic clusters PAM or PAL, while coexpression was observed in the SOG (see inset). (C) In the posterior brain section no co-expression was observed in dopaminergic clusters PPL1 or PPL2, but there was co-expression of GFP and TH in cluster PPM1/2 (see inset). There were also two neurons near the dopaminergic PPM1/2 cluster that were GFP specific, and had no TH expression (see inset, white arrow). In A-C size bar is equivalent to 100 μm. (D-G) Black scale bar, 1mm. (H-P) white scale bar, 0.5 mm. Bregma levels and described brain regions are according to Allen Mouse Brain Atlas. (H, I) Cortex layers 1–6. (J) basolateral amygdaloid nucleus, anterior part (BLA), basolateral amygdaloid nucleus, ventral part (BLV), basomedial amygdaloid nucleus, posterior part (BMP), central nucleus of amygdala, lateral part (CeL), dorsal endopiriform claustrum (DEn), stria medullaris (STIA), ventral endopiriform claustrum (VEn), (K) ventromedial thalamic nucleus (VM), (L) dorsomedial hypothalamic nucleus, ventral part (DMV), nigrostriatal tract (ns), ventromedial hypothalamic nucleus, central part (VMHC), ventromedial hypothalamuc nucleus, dorsomedial part (VMHDM), ventromedial hypothalamic nucleus, ventrolateral part (VMHVL), (M) Cortex layers 1–6, (N) parabrachial pigmented nucleus of the VTA (PBP), ventral tegmental area (VTA), (O) field CA1 of the hippocampus (CA1), field CA2 of the hippocampus (CA2), field CA3 of the hippocampus (CA3), lacunosum moleculare layer of the hippocampus (LMol), pyramidal layer of the hippocampus (Py), (P) crus 1 of the ansiform lobule (Crus1), lobule 4 and 5 of the cerebellar vermis (4/5Cb).

Next, to see where Ets96B could be involved in nervous system signalling we crossed *Ets96B-GAL4* to *UAS-GFP* and mapped GFP expression in the adult brain. Since dopamine is known to be a major regulator of the startle-response, we co-stained the brains for Tyrosine hydroxylase (TH), which is necessary for the production of L-DOPA and specifically marks dopaminergic neurons [37]. Interestingly we saw that Ets96B was almost exclusively expressed in dopaminergic neurons (Fig 7A–7C). Specifically, we saw Est96B-GAL4 induced GFP expression in the suboesphageal ganglionic (SOG) region in the brain (Fig 7A and 7B). Furthermore, Est96B-GAL4 induced GFP-expression co-localized with TH in a subset of dopaminergic PPM1/2 neurons (Fig 7C). Interestingly, within the PPM1/2 neurons there were two neurons that were GFP positive but TH negative (Fig 7C, inset, white arrows). There was also a strong overlap between Ets96B-GAL4 driven GFP and TH in the eye and the ventrolateral protocerebrum (VLP) (Fig 7A). Although GAL4 driven GFP is not an entirely reliable source for mapping gene expression, this result is an indication that *Ets96B* may be expressed in the regions of the *Drosophila* brain governing locomotion, as well as odour and visual-driven memory and learning. Furthermore, the *Ets96B-GAL4* driver may not recapitulate the entire *Ets96B* expression pattern.

In order to specify if the expression pattern of *Etv5* was enriched in dopaminergic-rich regions of the mouse brain, we performed detailed mRNA *in situ* hybridization mapping on coronal sections (Fig 7D–7G, S1 Table). *Etv5* mRNA was expressed in a specific and distinguishable pattern in certain brain regions, predominantly the cerebral cortex (Fig 7H, 7I and 7M), the amygdala (Fig 7J), and the hypothalamus (Fig 7L). Within the cerebral cortex there was conspicuously high expression in cortical layers 2, 4 and 6, while expression was almost absent from layers 1, 3, and 5 (Fig 7H, 7I and 7M). Furthermore, high expression was also observed in the dorsal and ventral endopiriform claustrum (Fig 7J). Strong expression was observed in limited parts of the amygdale, including the basolateral amygdaloid nucleus, ventral part (Fig 7J). Considerably high expression was found in the bed stria terminalis and the central amygdaloid nucleus (Fig 7J). Etv5 expression was observed at low to moderate levels in the hypothalamus, whereas expression was almost absent in the thalamus (Fig 7K and 7L). In conclusion, hypothalamic expression was found in the dorsomedial hypothalamic nucleus and also the central, dorsomedial and ventrolateral part of the ventromedial hypothalamic nucleus (Fig 7L). Strong expression was also identified in the nigrostriatal tract (Fig 7L). In the mesencephalon expression was restricted to the ventral tegmental area (VTA), including the parabrachial pigmented nucleus of the VTA (Fig 7N). In the hippocampus, Etv5 was strongly expressed in the pyramidal cell layer, CA1 through CA3. Some expression was also found in the lacunosum moleculare layer (Fig 7O). Low levels of *Etv5* mRNA expression were observed in the cerebellum, including the Granular, Molecular and Purkinje cell layers (Fig 7P). Other regions in the brain are listed in S1 Table.

### Ets96B required in dopaminergic neurons for proper metabolism and behaviour

**Fig 8:**
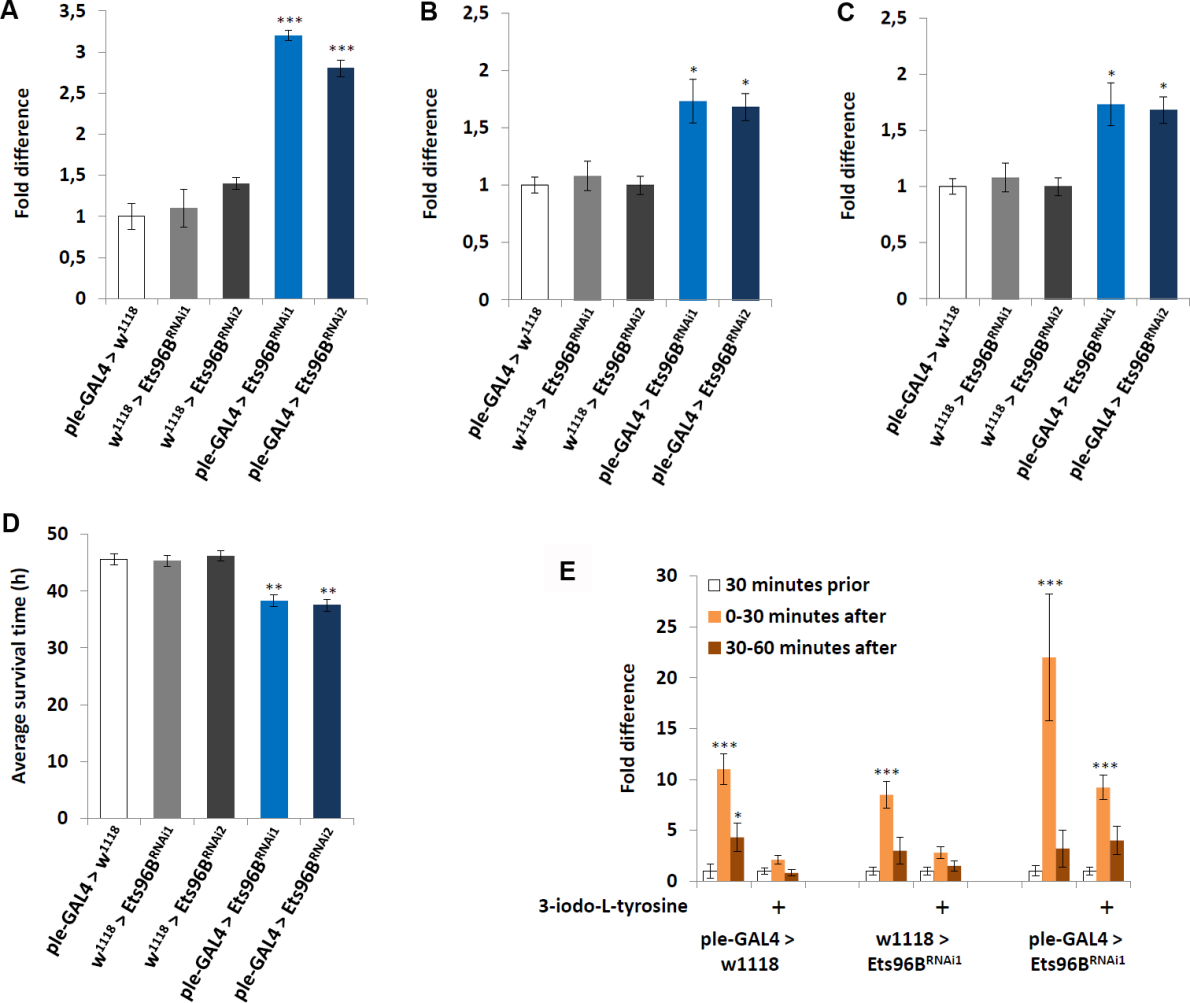
*Ets96B* required in dopaminergic neurons. (A-C) *Ets96B* was knocked down throughout development (*Ets96B*^*RNAi1*^ or *Ets96B*^*RNAi2*^) specifically in dopaminergic neurons using the *ple-GAL4* driver and the transcript level of genes (A) *ple*, (B) *Vmat* and (C) *DAT* was measured. RNA was collected from the heads of 5- to 7-day-old males for each genotype. qPCR was repeated at least 7 times for each transcript. (n = 25 males per treatment; * P<0.05, *** P<0.005 compared with controls, one-way ANOVA with Tukey’s post hoc test for multiple comparisons) (D) *Ets96B* was knocked down throughout development (*Ets96B*^*RNAi1*^ or *Ets96B*^*RNAi2*^) specifically in dopaminergic neurons using the *ple-GAL4* driver. 5–7 day old control and *Ets96B* knockdown males were placed in a vial containing 1% agarose and maintained at 25°C, DAMS was used to monitor activity. (n = 30–60 flies per genotype, ** = P < 0.01, one-way ANOVA was used to determine significance with Tukey’s post hoc test for multiple comparisons). (E) The DAMS system was used to monitor locomotion prior to and after light stimulation. Only *Ets96B*^*RNAi1*^ was used for this assay. (n = 30 males per strain; * P<0.05, *** P<0.005 compared with controls, Kruskal-Wallis non-parametric ANOVA was used to determine significance). In all graphs error bars = SEM.

Given that Ets96B and mouse Etv5 seemed to be expressed in dopaminergic-rich regions, we used the *ple-GAL4* driver (*pale* [*ple*] is the *Drosophila* tyrosine hydroxylase, necessary to convert tyrosine to L-DOPA) to knockdown *Ets96B* transcript specifically in dopaminergic neurons throughout development. We then examined the transcription levels of a select number of genes-*ple*, *Vesicular monoamine transporter* (*Vmat*), and *Dopamine transporter* (*DAT*)-known to influence dopamine production, release or reuptake (Fig 8A–8C). Transcription levels of the *ple-GAL4* heterozygous controls were set at 100%, shown as 1 on the various graphs. When *Ets96B* expression was knocked down in dopaminergic neurons, the transcript level of *ple* was significantly upregulated (P < 0.005) (Fig 8A). Furthermore, the expression of *Vmat*, involved in the transportation of monoamines such as dopamine into the synaptic vesicles, was also increased in *Ets96B* knockdown males (P < 0.05) (Fig 8B). Finally, the expression of *DAT*, which is necessary to pump dopamine out of the synapse back into cytosol, was significantly lower when *Ets96B* expression was knocked down in dopaminergic neurons (P < 0.005) (Fig 8C). Increased dopamine production and decreased dopamine reuptake should lead to prolonged dopamine signalling.

Next, we asked if knocking down *Ets96B* specifically in dopaminergic neurons was sufficient to recapitulate the starvation and startle-response phenotypes. Similar to what was observed using the *elav-GAL4* and *Ets96B-GAL4* drivers, knocking down *Ets96B* specifically in dopaminergic neurons throughout development made adult males more susceptible to starvation (P < 0.01) (Fig 8D). Furthermore, lowering *Ets96B* transcript levels in dopaminergic neurons was sufficient to replicate the heightened light-induced startle-response (P < 0.005) (Fig 8E). To determine if the increased startle-response observed when *Ets96B* was knocked down specifically in dopaminergic neurons was due to increased dopamine signalling, we fed adult males the tyrosine hydroxylase inhibitor 3-iodo-L-tyrosine before performing the light-induced startle-response assay. In controls this was sufficient to abolish the light-induced startle-response (Fig 8E). In males where *Ets96B* was knocked down in dopaminergic neurons, inhibiting dopamine production reduced the startle-response to the same level as normal fed controls (Fig 8E). From this result we conclude that reducing *Ets96B* expression in dopaminergic neurons increases dopamine signalling.

### Human ETV5 SNPs link to BMI and Bipolar disorder

**Fig 9:**
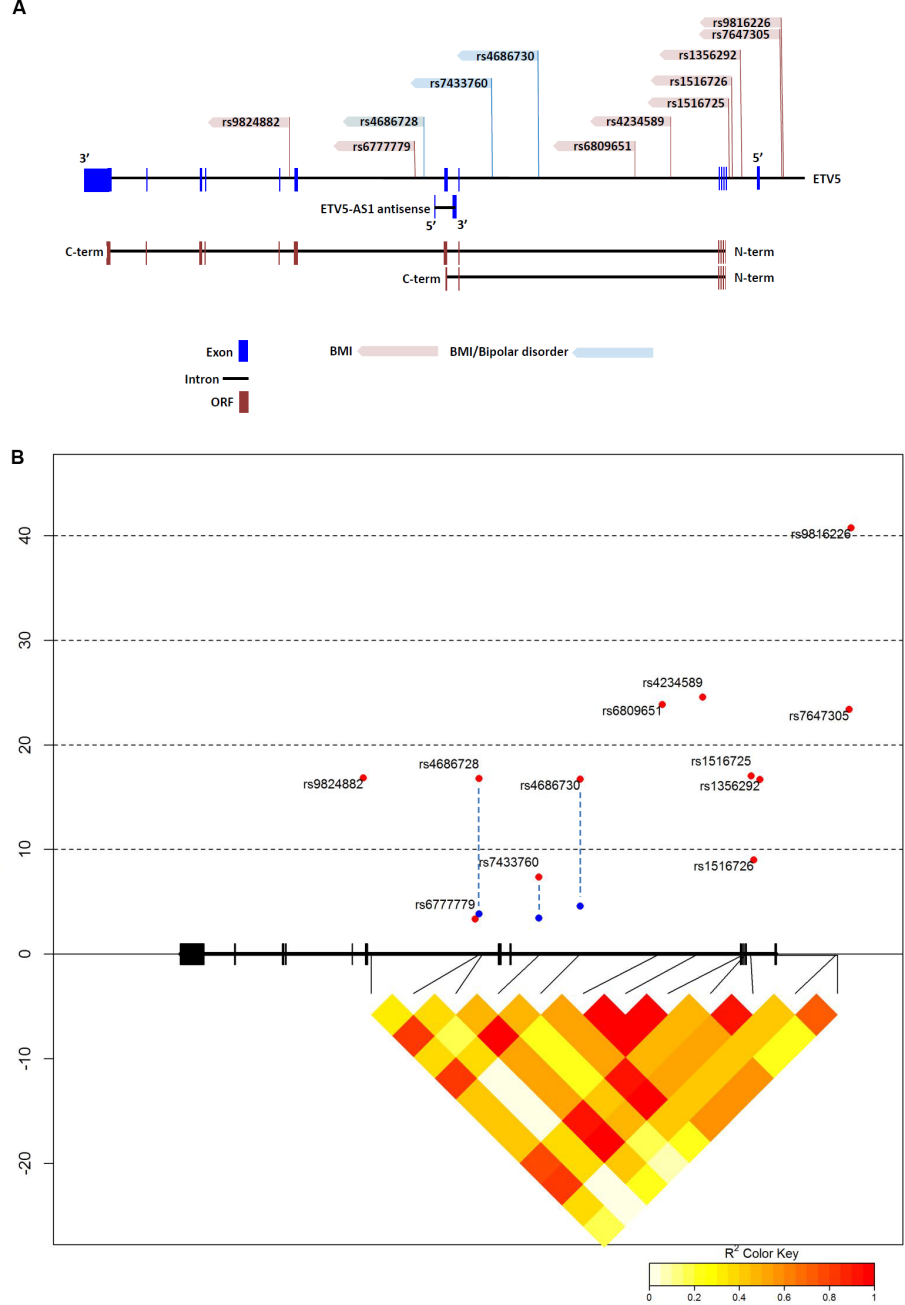
Human *ETV5* SNPs associated with body mass index and bipolar disorder. (A) Localization of relevant single nucleotide polymorphisms (SNPs) in the ETV5 gene. SNP names according to NCBI dbSNP. The GWAS central database was used to link ETV5 associated SNPS to BMI. Interestingly, three SNPs were also associated with bipolar disorder (blue SNPs). The three bipolar-linked SNPs all localize around exons 6 and 7, which is also the location of the long, noncoding RNA (lncRNA) ETV5-AS1. Snap Proxy was then used to determine Pair-linked disequilibrium in various populations. (B) Linkage disequilibrium between SNPs in ETV5. Black boxes = exons, thick black lines between boxes = introns, red points = SNPs associated with BMI in GWAS, blue points = SNPs associated with bipolar disorder in GWAS. The LD between SNPs in the CEU population is expressed as R^2^ and displayed as a color code below the plot.

The startle-response phenotype demonstrated by loss of *Ets96B* in the nervous system is similar to the effect of prepulse inhibition (PPI) in mammals. PPI is a neurological phenomenon where a weaker prestimulus (prepulse) inhibits the reaction to a subsequent stronger startle stimulus (pulse). In humans disrupted PPI responses are linked with bipolar disorder [38]. Using the GWAS Central database [39] we searched for *ETV5* localized SNPs that had a significant link (P < 0.05) to either BMI or bipolar disorder. Employing this method we found 12 SNPs mapped to *ETV5* that significantly linked to BMI (S2 Table). Interestingly, three of these SNPs were also significantly linked to bipolar disorder (S2 Table). Next, we made a map to define where these 12 SNPs localized within the *ETV5* genomic region (Fig 9A). Of notable interest, the three BMI/bipolar associated SNPs all localized around *ETV5* exons 6 and 7 (Fig 9A, blue highlighted SNPs). This is an interesting genetic region as there is evidence a truncated ETV5 transcript is produced that stops after exon 7, which would encode only the inhibitory N-terminal PEA3 domain [40]. Furthermore, this same region encoded a long non-coding antisense RNA (lncRNA) called *ETV5-AS1* (Fig 9A). Using the NONCODE database [41] we determined that *ETS5-AS1* was expressed in the adrenal glands, testis and thyroid.

Using Encode Roadmap {http://www.encode-roadmap.org/} (S4 Fig) and RegulomeDB [42] (Table 2) databases we determined the methylation state of the *ETV5* gene in various brain regions. By means of these databases we found that SNPs rs7647305 and rs1356292 mapped to the promoter region. In fact, rs7647305 was predicted to map to the TATA-box and rs1356292 was predicted to be at the transcription start site (Table 2). It was interesting that rs4686730 was predicted to map to an insulin promoter factor 1 (IPF1) binding site (Table 2), considering that *Etv5* was found to be necessary for the secretion of insulin in mice [43]. When examining the chromatin state of the three SNPs linked to BMI and BD, (rs4686730, rs7433760 and rs4686728) they were all quiescent in the brain germinal matrix (BGM) (Table 2). Furthermore, in brain regions known to control reward response, such as the anterior caudate (BAC) and midfrontal lobe (BMFL), rs7433760 mapped near a region with H3K4 single methylation, indicating an active enhancer (S4 Fig). In the RegulomeDB database, rs7433760 was predicted to map to an active enhancer in the angular gyrus (BAG), dorsolateral prefrontal cortex (BDPF) and the hippocampus middle (BHM), areas involved in decision making and memory. Finally, we made a linkage disequilibrium map that demonstrated tight linkage between all *ETV5* SNPs linked to BMI and BD, except for BMI-linked rs6777779 (Fig 9B).

**Table 2:** Regulatory elements and chromatin states associated with *ETV5* SNPs.

		Histone Modifications/Chromatin State									
rsID	Bound Protein/Motifs	BAG	BAC	BCG	BDPF	BGM	BHM	BITL	BSN	FB-F	FB-M
**rs9816226**	ND	ND	ND	ND	ND	ND	ND	ND	ND	ND	ND
**rs7647305**	Pit1, Tbp	Q/L	WRPC	Q/L	Q/L	WRPC	WT	Q/L	WT	Q/L	WRPC
**rs1356292**	None	FATSS	FATSS	FATSS	FATSS	FBTSS	FATSS	ATSS	E	ATSS	FSTSS
**rs1516726**	ND	ND	ND	ND	ND	ND	ND	ND	ND	ND	ND
**rs1516725**	GR	WT	WT	WT	WT	RPC	E	WT	WT	WT	WRPC
**rs4234589**	FOX	WT	WT	WT	WT	WT	WT	WT	WT	E	FATSS
**rs6809651**	ND	ND	ND	C	ND	ND	ND	ND	ND	ND	ND
**rs4686730**	MAX/ER, c-Myb, OPF1. TGIF1	WT	WT	WT	WT	Q/L	WT	WT	WT	Q/L	WT
**rs7433760**	None	E	WT	WT	E	Q/L	E	WT	WT	Q/L	WT
**rs4686728**	None	WT	WT	WT	WT	Q/L	WT	WT	WT	WT	WT
**rs6777779**	ND	ND	ND	ND	ND	ND	ND	ND	ND	ND	ND
**rs9824882**	HNF3beta, Oct1, Fox	WT	WT	ST	WT	WT	ST	ST	WT	WT	St

Abbreviations: POU domain, class 1, transcription factor 1 (Pit1), TATA-binding protein (Tbp), glucocorticoid receptor (GR), Forkhead box (Fox), MYC associated factor X (MAX), Insulin Promoter Factor 1 (IPF1), TGFB-Induced Factor Homeobox 1 (TGIF1), hepatic nuclear factor 3 beta (HNF3beta), Octamer-Binding Protein 1 (Oct1), No data (ND), quiescent, low transcription (Q/L), weak transcription (WT), strong transcription (ST), weak repressed polycomb (WRPC), active transcription start site (ATSS), flanking active transcription start site (FATSS), flanking bivalent transcription start site (FBTSS), enhancer (E).

**Fig 10:**
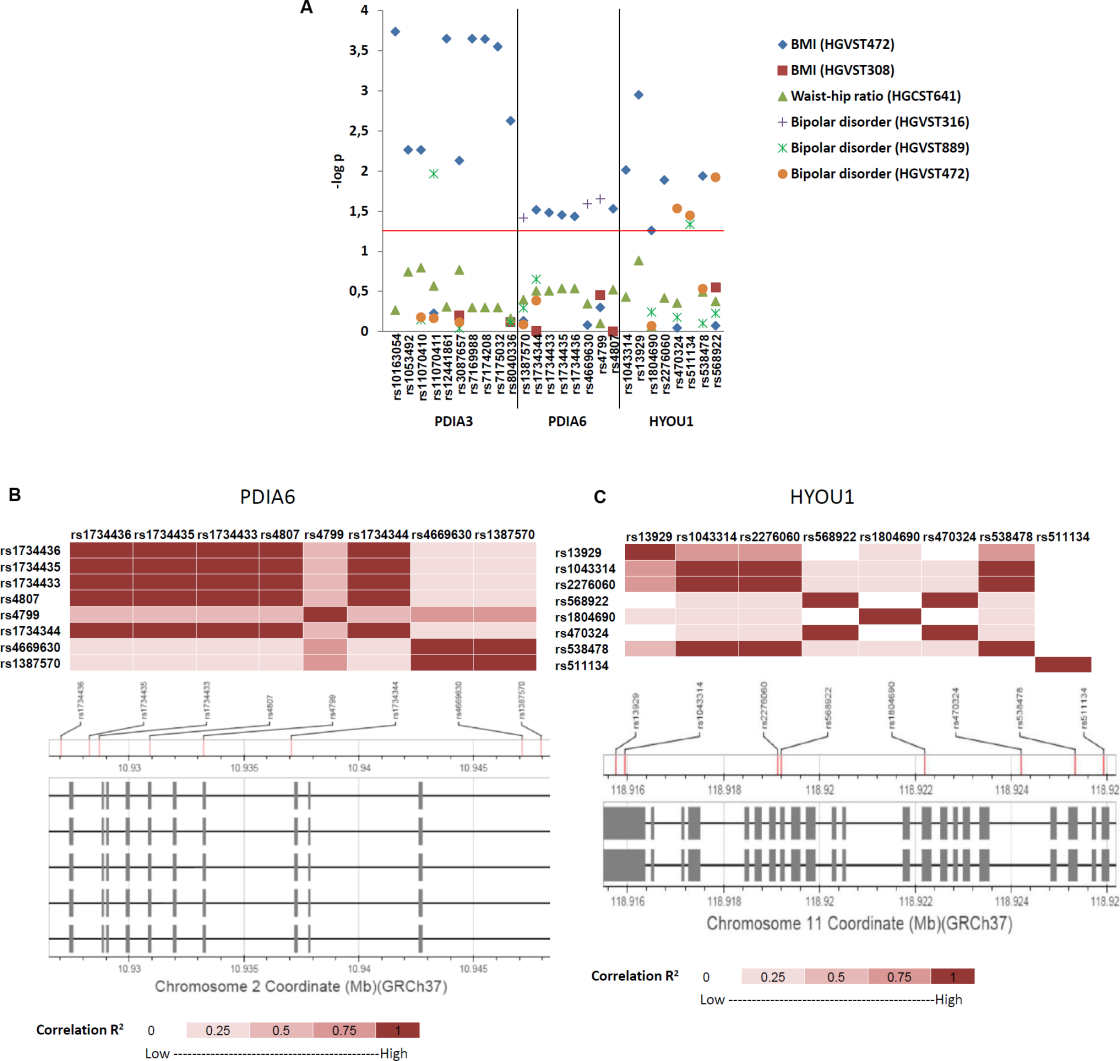
Human ER molecular chaperon SNPs associated with body mass index and bipolar disorder. (A) Summary figure of SNP association results from Genome Central [39] for *PDIA3*, *PDIA6* and *HYOU1* linkage to body mass index (BMI), Weight-hip ratio and bipolar disorder. Horizontal axis lists significant SNPs and genes. The red line represents P = 0.05 nominal significance level; above the line represents significant results. (B) Linkage disequilibrium between SNPs in PDIA6. Boxes = exons, lines between boxes = introns, The LD between SNPs in the CEU population is expressed as R^2^ and displayed as a color code below the plot. (C) Linkage disequilibrium between SNPs in HYOU1. Boxes = exons, lines between boxes = introns, The LD between SNPs in the CEU population is expressed as R^2^ and displayed as a colour code below the plot.

Next we wanted to determine if the human homologues of the *Drosophila* ER molecular chaperones (Fig 3D), whose expression is regulated by *Ets96B*, also linked to BMI and BD. Using data from multiple GWAS studies (BMI, Waist-hip ratio or bipolar disorder [8, 44–46]) we searched for SNPs localizing to the human ER molecular chaperone homologues. We found SNPs with a significant link (P < 0.05) to either BMI or BD that localized to three of the homologues (*PDIA3*, *PDIA6* and *HYOU1*) (Fig 10A). We located 9 SNPs mapped to *PDIA3*, 5 SNPS mapped to *PDIA6* and 5 SNPS mapped to *HYOU1* that significantly linked to BMI. Furthermore, we found 1 SNP mapped to *PDIA3*, 3 SNPs mapped to *PDIA6* and 3 SNPs mapped to *HYOU1* that significantly linked to BD (Fig 10A). Next, we made linkage disequilibrium maps for *PDIA6* and *HYOU1* that demonstrated tight linkage between all *PDIA6* SNPs linked to BD (Fig 10B); the same was true for *HYOU1*, except for BD-linked rs511134 (Fig 10C).

## Discussion

We knocked down the *Drosophila ETV5* homolog *Ets96B* in neurons and discovered that it regulates expression of genes involved in the mitochondrial electron transport chain (See Table 1), as well as endoplasmic reticulum localized molecular chaperones. Furthermore, we observe that loss of *Ets96B* affects starvation resistance, lipid storage, startle-response and sleep/wake behaviour. Moreover, knocking down Ets96B specifically in dopaminergic neurons influences the expression of genes involved in dopamine signalling, and is sufficient to recapitulate both metabolic and behavioural phenotypes. Finally, we map human *ETV5*-associated SNPs linked to BMI and bipolar disorder to a location surrounding *ETV5* exons 6 and 7. This region is interesting as it may be involved in regulating the expression of a truncated ETV5 protein, comprising only the PEA3 inhibitory domain [40]. This same region also contains a long non-coding RNA (lncRNA), *ETV5-AS1*, whose function is unknown at this time; however, there is evidence that lncRNAs regulate alternative splicing [47].

The involvement of *ETV5* homologues in the development and maintenance of the dopaminergic system has been studied previously [48–50]. The first evidence of an *ETV5* homologue regulating dopaminergic neuronal development was discovered in *C*. *elegans*, where loss of *ast-1* resulted in the failure of dopaminergic neurons to develop [48]. Subsequently, dopaminergic neuronal development in the substantia nigra (SN) and ventral tegmental area (VTA) of *Etv5* knockout mice was studied. In *Etv5* knockout mice, within the studied regions the dopaminergic system developed normally and there was no significant difference observed in genes that regulate dopamine signalling, such as *dopamine transporter* (*Dat*) or *vesicular monoamine transporter 2* (*Vmat2*) [49]. Our study demonstrates that Etv5 is extensively expressed throughout the mouse brain (see Fig 7G–7P). One region where we observed high levels of Etv5 expression, the amygdala (see Fig 7J), was not studied in Wang and Turner [49]. Interestingly, in mice dopaminergic neurons within the amygdala have been shown to regulate both anxiety and depressive-like behaviours [51, 52]. There is a possibility that Etv5 function in the regulation of dopaminergic neuronal development or maintenance is more important in areas other than the SN or VTA, such as the amygdala. Given that we show that *Drosophila* Ets96B regulates genes involved in cellular stress, in mice Etv5 might only become important in dopaminergic neurons when they come under stress. These possibilities should be followed up in future studies. Finally, more recently it was discovered that *etv5* co-expressed with tyrosine hydroxylase in the brain of a cichlid fish, *Astatotilapia burtoni*, but no functional analysis was undertaken in this study [49].

Our study indicates that in *Drosophila* Ets96B is necessary to inhibit dopamine signalling. In fact, our results indicate that in the fruit fly *Ets96B* acts to repress the expression of more genes that it activates. Possibly, there are co-factors that interact with Ets96B to govern if it is a transcriptional repressor or activator. There is evidence for this with other Ets-family members. For instance, in both mammals and *Drosophila*, Ets-1 (known as Pointed in *Drosophila*) can act as an activator or inhibitor depending on interactions with Runx1 (known as Lozenge in *Drosophila*) [53, 54]. Depending on temporal or spatial differences, this interacting protein may or may not be present, meaning in some tissues or conditions Ets96B would act as a transcriptional activator, while under other conditions it would act as a repressor. Given that our whole transcriptome sequencing was performed on material collected from well fed, equally aged, whole male adults, our results cannot determine if this is the case.

The largest group of genes affected by loss of *Ets96B* throughout development in the *Drosophila* nervous system is involved in oxidation/reduction, including those regulating mitochondrial electron transport (see Table 1). Interestingly, many studies have linked the mitochondrial electron transport chain to obesity, as well as insulin resistance [55–58]. This includes *COX1* and *CYTB*, human homologues of two genes (*mt*:*Col* and *mt*:*Cyt-b*) overexpressed when *Ets96B* is knocked down in the CNS, which are linked to insulin sensitivity [59, 60]. Lower expression levels of these two genes correlates with insulin resistance [60]. Furthermore, an association with obesity was found for a frequent allele of the human *ATP6* gene [61, 62]. Similar to *mt*:*Col* and *mt*:*Cyt-b*, the *Drosophila* homolog of *ATP6*, *mt*:*ATPase6*, is also upregulated when *Ets96B* is knocked down in the CNS.

When *Ets96B* is knocked down in the nervous system during development a group of conserved interacting endoplasmic reticulum (ER) proteins are highly upregulated: *CaBP1*, *Crc* and *ERp60* (see Fig 3A and 3B). All three proteins localize to the ER, where they are involved in the regulation of protein folding. Also, human homologues of *CaBP1* and *ERp60*, known as *PDIA6* and *PDIA3*, are involved in disulfide bridge formation (see Fig 3E). Of note, insulin contains three important disulphide bonds that are conserved in *Drosophila* insulin-like peptides, and recently it was demonstrated that *Etv5* knockout mice are deficient in insulin secretion [43], though not due to a decrease in insulin production. Disulphide bonds are important for proper protein folding, secretion and function, and disruption of disulfide bond formation in insulin proteins could inhibit proper secretion.

Previously, it was demonstrated that *Crc* mutant flies fail to react when startled [36, 63]. Interestingly, we observed that adult males have a heightened startle-response when *Ets96B* is knocked down in the CNS during development, but when *Ets96B* expression is specifically knocked down in adults there is no difference in activity compared to control flies. Furthermore, compared to controls, knocking down *Ets96B* expression throughout development induces hyperactivity. When *Crc* was mutated, the mushroom bodies and ellipsoid body were smaller than normal. *Ets96B* is also expressed in the mushroom bodies and the ellipsoid body of the *Drosophila* CNS [32]. The mushroom bodies are the centre of learning and memory [64] while the ellipsoid body mediates stress-related locomotion [65, 66], and both regions are important components of the dopamine and serotonin systems [65, 66]. In *Drosophila* the ellipsoid body regulates the startle-response; more specifically, dopaminergic neurons within the ellipsoid body regulate the arousal-response to being startled [67]. Possibly, through its regulation of the molecular chaperon complex, *Ets96B* controls the proper development or signalling of dopaminergic neurons within the mushroom bodies and the ellipsoid body. Interestingly, when murine dopaminergic neurons are put under stress they upregulate the *Crc* homologue *Calreticulin* (*Calr*) [20, 21]. Furthermore, rat brains, exposed to neurotoxic 6-hydroxydopamine, induce the expression of not only *Calr*, but also *Pdia3* (*Drosophila ERp60*) [68]. Furthermore, in *C*. *elegans*, CRT-1 (Calr) was necessary for regenerative neuronal outgrowth from neuronal injury [69]. This leads to the possibility that lowering *Ets96B* expression throughout development is neuroprotective and actually leads to increased dopaminergic signalling in adults due to increased dopaminergic neuronal innervations. In regions of the human brain linked to bipolar disorder, such as the anterior caudate and mid-frontal lobe [70], the ETV5-linked SNP rs7433760 mapped to an enhancer specific for those regions (see S4 Fig). Thus, a decrease in *ETV5* expression could lead to increased dopaminergic signalling.

When *Ets96B* is knocked down in the nervous system during development, phenotypes mimicking obesity (increased lipid storage) and bipolar disorder (startle-response and disrupted sleep) are observed. Of note, we found 12 *ETV5* localized SNPs that link to BMI, of which three also link to BD (see Fig 9, as well as S2 Table and S4 Fig). Intriguingly, *HSP90B1* (*Drosophila Gp93*) and *CALR* (*Drosophila Crc*), homologues of molecular chaperones upregulated when *Ets96B* was knocked down in the nervous system, are not properly induced during increased endoplasmic reticulum (ER) stress in lymphoblastoid cells recovered from bipolar disorder patients [18]. Furthermore, we also found multiple SNPs that localize to *PDIA3*, *PDIA6* and *HYOU1* that link to either BMI or bipolar disorder (see Fig 10A).

Another possibility of how disruption of ER localized molecular chaperone expression may link to obesity and/or BD is increased expression of vesicle monoamine transporter 2 (VMAT2), which concentrates monoamine neurotransmitters into synaptic vesicles. Our results show that loss of *Ets96B* in dopaminergic neurons is sufficient to increase tryosine hydroxylase (*ple*) *Vmat* expression and decrease *DAT* expression, which would lead to increased dopamine signalling. Mammalian VMAT2 contains a disulphide bridge in its vesicular luminal loops that contributes to efficient monoamine transport [71]. This could be enhanced by increased expression of *PDIA3*, *PDIA6* or *CALR*, whose *Drosophila* homologues, *CaBP1*, *ERp60* and *Crc*, were all upregulated when *Ets96B* was knocked down in the nervous system. Increased VMAT2 function would influence monoamine signalling, including dopamine and serotonin. Vmat2 expression levels are reduced in Flinders sensitive line (FSL) rats, which represent a genetic animal model for clinical depression in humans [72, 73]. This suggests that alterations in VMAT2 may play a role in the aetiology of depression and anxiety. Interestingly, reduced Vmat2 levels were localized to the ventral tegmental area and the substantia nigra in FSL rats, regions that express *Etv5* in mice [9, 73]. Interestingly, in FSL rats, the reduced levels of Vmat2 protein were not caused by a reduction in *Vmat2* mRNA levels. Thus, the activity of Vmat2 in FSL rat brain is likely not affected at the transcriptional level, but at the functional level.

In conclusion, here we identify a specific molecular link between obesity and bipolar disorder, both conditions with highly complex and overlapping genetics and pathophysiology. We found that Ets96B regulates the expression of cellular systems with links to obesity and behaviour, with phenotypes at the physiological level in the mutant flies supportive of these findings. In particular, we find that *Ets96B* inhibits the expression of a conserved ER molecular chaperone complex shown to be neuroprotective in dopaminergic neurons. Furthermore, Ets96B is expressed in a subset of dopaminergic neurons in adult males and loss of *Ets96B* specifically in dopaminergic neurons influences the expression of genes involved in dopamine production, release and reuptake and would increase dopamine signalling. Finally, the new association of *ETV5* to human bipolar disorder emphasizes a functional relationship between obesity and BD at the molecular, as well as behavioural level.

## Methods

### Fly stocks and maintenance

w*, *P{w[+mW.hs]=GawB}elav[C155]*, *w*^*1118*^*;P{y[+t7*.7] w[+mC] = GMR36H05-GAL4}attP2 P{GawB}elavC155 w*; P{FRT(whs)}G13 P{tubP-GAL80}LL2, and the w*; P{w[+mC] = ple-GAL4.*F}3* driver lines, the w*; UAS-GFP line, as well as the Transgenic RNAi Project (TRiP) [25] RNAi lines *y*^*1*^,sc*,v^1^;*P{TRiP*.*HMS00092}attP2* (*Ets96B*^*RNAi1*^) and *y1*, v1; *P{TRiP*.*JF02226}attP2* (*Ets96B*^*RNAi2*^) and the RNAi lines *w*^*1118*^*;P{w**[+mC] = UAS-GFP*.*dsRNA}143* (*GFP*^*RNAi1*^) and *w*^*1118*^;*P{w[+mC] = UAS-GFP*.*dsRNA}142* (*GFP*^*RNAi2*^) [74] were received from the Bloomington Stock Center (Bloomington, Indiana, USA; http://flystocks.bio.indiana.edu/). All strains were crossed into the same *w*^*1118*^ genetic background. All flies, unless otherwise stated, were maintained on Jazz mix standard fly food (Fisher Scientific, Göteborg, Sweden), supplemented with 5% Brewer’s yeast extract (VWR, Stockholm, Sweden). Flies were maintained at 25°C in an incubator at 60% humidity on a 12:12 light:dark cycle. Flies crossed to GAL4 drivers (GAL4 drivers crossed to UAS-RNAi lines) and controls (GAL4 drivers or UAS-RNAi crossed to *w*^*1118*^) were raised at 25oC until the adults emerged (F_1_ generation); once collected adults were raised at 29oC for the appropriate times. All assays were performed at 25°C. In all assays, the GAL4 drivers and UAS transgenic flies were crossed to *w*^*1118*^ flies and their F1 progeny used as controls.

### Multiple sequence alignments and Phylogenetic analysis

The protein sequences used for the sequence alignment and phylogenetics analysis were mined using Flybase (www.flybase.org), NCBI HomoloGene (www.ncbi.nlm.nih.gov/homologene), and Hmmer (www.ebi.ac.uk/Tools/hmmer/). Protein sequences were aligned using CLC sequence Viewer 6 using default parameters. The sequence alignment used for phylogenetic analysis was exported in clustal format and viewed using MEGA5 [75]. The file was then transformed into a MEGA file for further analysis. Evolutionary history was inferred using the Maximum-likelihood method. The optimal tree with the sum of branch length = 3.794 is shown. The percentage of replicate trees in which the associated taxa clustered together in the bootstrap test (5000 replicates) is shown next to the branches. The tree is drawn to scale, with branch lengths in the same units as those of the evolutionary distances used to infer the phylogenetic tree. The evolutionary distances were computed using the Dayhoff matrix based method and are in the units of the number of amino acid substitutions per site. The rate variation among sites was modelled with a gamma distribution (shape parameter = 5). The analysis involved 35 amino acid sequences. All positions containing gaps and missing data were eliminated. There were a total of 170 positions in the final dataset. Evolutionary analyses were conducted in MEGA5 [75].

### Library preparation and sequencing

#### Primary processing of SOLiD RNA-Seq reads

RNA-seq reads for whole transcriptome were obtained using SOLiD 5500xl paired end sequencing from life technologies. This version produces read length of 75 bp for fragment libraries with the alternative to sequence an additional 35 bp in the reverse direction (paired-end sequencing). The samples were divided into six libraries and since the libraries were molecularly bar-coded the separation of libraries for control and experimental samples was done effortlessly using respective barcodes. The initial quality analysis was performed using a propriety tool 'XSQ Tools package' provided by the life technologies. This package also provides tools for converting files from XSQ to csfasta format and additionally provides qual files containing read quality information. Further analysis was done using the 'Tuxedo suit' [76] composed mainly of three tools TopHat, Cufflinks and CummRbund.

#### Mapping of RNA-Seq reads using TopHat

TopHat (v2.0.6) incorporates an ultra high-throughput short read aligner Bowtie (version 0.12.7) [77] as alignment engine. Based on provided quality control information TopHat removes a portion of reads and maps the remaining reads to reference genome. Reads were then aligned to the *D*. *melanogaster* reference genome (build dmel_r5.47_FB2012_05) obtained from flybase using TopHat using the prebuilt bowtie index downloaded from the TopHat homepage (http://TopHat.cbcb.umd.edu/igenomes.shtml).

#### Transcript assembly and abundance estimation using Cufflinks

The aligned reads were then processed by Cufflinks v2.0.2. Cufflinks tool estimates the relative abundances of transcripts based on how many reads support each transcript, taking into account biases in library preparation protocols and reports it in “fragments per kilobase of transcript per million fragments mapped” or FPKM. Cufflinks constructs a minimum set of transcripts describing the reads in the dataset rather than using existing gene annotation allowing Cufflinks to identify alternative transcription and splicing. However it should be noted that Cufflinks (and other tools too) are dependent on the provided genome annotation and therefore the reported FPKM values relate only to genes described and genes missing in the annotation description file (even though reads map to it) will not be reported.

#### Differential expression testing using Cuffcompare and Cuffdiff

Cuffcompare was used to produce combined General Transfer Format (GTF) files of the six libraries. The GTF files were then passed to cuffdiff along with original alignments obtained with TopHat. Cuffdiff re-estimates the abundance of transcripts listed in.GTF files using alignment files. The differential expression is checked for genes, transcripts and isotopes. By tracking changes in the relative abundance of transcripts with a common transcription start site, cuffdiff can identify changes in splicing. We used cuffdiff to compare expression difference between three stages of controls and one experimental sample wherein gene *ETS96B* was down regulated. Cuffdiff learns how read counts vary for each gene across the replicates and uses these variance estimates to calculate the significance of observed changes in expression. The calculated P value and q value (the FDR-adjusted P value of the test statistic) from cuffdiff were used to determine significance of differential expression The significance depends on whether P is greater than the false discovery rate (FDR) after a Benjamini-Hochberg correction [78] for multiple testing

### RNA purification

The phenol-chloroform method was used for RNA extraction from tissue samples [79]. Fifty fly heads were homogenized with 800 μl TRIzol (ThermoFisher Scientific, MA, USA), 200 μl Chloroform (Sigma-Aldrich, MO, USA) was added and samples were centrifuged at 12000 rpm for 15 minutes at 4°C. The aqueous layer, which contained RNA, was separated and 500 μl isopropanol (Solvaco AB, Sweden) was added. The RNA was precipitated by storing the samples at -32°C for 2 hours. Samples were centrifuged at 12000 rpm for 10 minutes at 4°C, to collect the RNA pellets, which were then washed with 75% ethanol (Solvaco AB, Sweden) to remove the organic impurities. Samples were allowed to air dry to remove any traces of ethanol. Dried RNA pellets were dissolved in 21.4 μl of RNAse free water (Qiagen GmBH, Germany) and 2.6 μl of DNAse incubation buffer (Roche GmBH, Germany). The samples were incubated at 75°C for 15 minutes to ensure complete dissolution of RNA-pellets. 2 μl of DNAse I (10 U/μl, Roche GmBH, Germany) was added to each sample, and incubated at 37°C for 3 hr to remove DNA contamination. DNAse was deactivated by incubating the samples at 75°C for 15 minutes. Removal of DNA was confirmed by PCR using Taq polymerase (5U/μl, Biotools B & M Labs, Spain), followed by agarose gel electrophoresis. The RNA concentration was measured using a nanodrop ND 1000 spectrophotometer (Saveen Werner).

### cDNA synthesis

cDNA was synthesized from RNA template using dNTP 20 mM (ThermoFisher Scientific, MA, USA), random hexamer primers and M-MLV Reverse Transcriptase (200 U/μl, ThermoFisher Scientific, MA, USA) by following manufactures instructions. cDNA synthesis was confirmed by PCR followed by agarose gel electrophoresis.

### qRT-PCR

Relative expression levels of three housekeeping genes (*EF-1*, *Rp49* & *RpL11*) and of the genes of interest were determined with quantitative RT-PCR (qPCR). Each reaction, with a total volume of 20 μl, contained 20 mM Tris/HCl pH 9.0, 50 mM KCl, 4 mM MgCl2, 0.2 mM dNTP, DMSO (1:20) and SYBR Green (1:50000). Template concentration was 5 ng/μl and the concentration of each primer was 2 pmol/μl. Primers were designed with Beacon Designer (Premier Biosoft) using the SYBR Green settings. All qPCR experiments were performed in duplicates; for each primer pair a negative control with water and a positive control with 5 ng/μl of genomic DNA were included on each plate. Amplifications were performed with 0.02 μg/ml Taq DNA polymerase (Biotools, Sweden) under the following conditions: initial denaturation at 95°C for 3 min, 50 cycles of denaturing at 95°C for 15 sec, annealing at 52.8–60.1°C for 15 sec and extension at 72°C for 30 sec. Analysis of qPCR data was performed using MyIQ 1.0 software (Bio-Rad, CA, USA) as previously reported [80]. Primer efficiencies were calculated using LinRegPCR [81] and samples were corrected for differences in primer efficiencies. The GeNorm protocol described by Vandesompele et al. [82] was used to calculate normalization factors from the expression levels of the housekeeping genes. Grubbs' test was performed to remove outliers. Differences in gene expression between groups were analyzed with ANOVA followed by Fisher's PLSD test where appropriate. P<0.05 was used as the criterion of statistical significance. The following primers were used: *EF-1* F: 5´-GCGTGGGTTTGTGATCAGTT-3´, R: 5´-GATCTTCTCCTTGCCCATCC-3´; *Rp49* F: 5´-CACACCAAATCTTACAAAATGTGTGA-3´, R: 5´-AATCCGGCCTTGCACATG-3´; *RpL11* F: 5´-CCATCGGTATCTATGGTCTGGA-3´, R: 5´-CATCGTATTTCTGCTGGAACCA-3´, *Ets96B* F: 5´-CCTCCATAGACTACCATAT-3´, R: 5´-GTAACTCAACCTCAATGC-3´; *CaBP1* F: 5´-GCAGCGTTAGTGCCTTCTATT-3´, R: 5´-CTTTCAGCACCTCCCGGTC-3´; *ERp60* F: 5´-GACTTTGCCACCACCCTAAAA-3´, R: 5´-TACTCGGGCTTCAATCGCTTG-3´; *Crc* F: 5´-GAAAACTGGGAGGATACGTGG-3´, R: 5´-GAGAGGTCTGAATGCCTTTGTC-3´; *ple* F: 5´-CGAGGACGAGATTTTGTTGGC-3´, R: 5´-TTGAGGCGGACCACCAAAG-3´; *Vmat* F: 5´-CGTGACCTTCGGGACGATAG-3´, R: 5´-ACTAGAGCGGGAAAACCAGC-3´; DAT F: 5´-GCTTCAAACCATAAGTTCTAA-3´, R: 5´-TCGGACTTGATATTATCTACAA-3´

### Starvation assay

Starvation resistance assay was performed in a similar fashion as Hergarden et al (2012) [33] Starvation resistance was measured by placing 30, 5 to 7 days old, male flies in individual tubes containing 1% agarose in the Drosophila activity monitoring system (DAMS) (Trikinetics, MA, USA). DAMS was performed at 25°C in an incubator, on a 12h:12h light:dark cycle.

### Triacylglycerol determination

Flies (25 males) were homogenized with 100 μl of PBST buffer (1x phosphate buffer with 10% Tween20), incubated at 70°C for 5 minutes and then centrifuged at maximum speed for 10 minutes. The supernatant was transferred into a new eppendorff and used as samples. A glycerol standard was used to generate a standard curve with concentrations of 1.0, 0.8, 0.6, 0.4, and 0.2 mg/ml equivalent triolein concentration. 100 μl of free glycerol reagent was added to 10μl of blank (PBST), standards or samples and initial absorbance at 540nm was measured after incubation at 37°C for 15 minutes. The concentration of free glycerol in the samples was calculated from the standard curve generated by this initial absorbance value. Then, 20 μl of triglyceride reagent was added in each standard and sample and incubated at 37°C for 15 minutes. The final absorbance was taken at 540 nm to calculate triglyceride concentration from the generated standard curve. The protein concentration of each sample was measured by the Bio-Rad (Hercules, CA, USA) protein assay kit. The concentrations of free glycerides and Triglycerides in samples (mg/mg of protein) were calculated from five replicates.

### Startle-response assay

We measured startle behaviour as described previously [50]. In brief, single 5–7 day old males were placed in a plastic culture vial and subjected to a mechanical disturbance by a gentle, yet sudden, tap of the vial. The vial was then placed horizontally on a bench top. Startle-induced locomotion was calculated as the number of seconds each fly was active within the 60 s period following the startle-induced locomotion. All tests were done at 25°C with 3 hours of lights on. The sample size for all genotypes was N = 50.

### DAMS activity assays

Flies were tested using the Drosophila Activity Monitoring System (DAM2, TriKinetics, www.trikinetics.com). Flies were placed in 5mm x 65mm glass tubes during the morning, with food placed at one end of the tube. Flies were continually monitored by the use of a light beam, with the number of beam breaks continuously recorded over a 48 hour period. A light shock or sound shock stimulus was given approximately one day after the animals were first placed in the tubes, at 12 noon. The number of beam breaks during the first four hours the flies were housed in the tube (when the arena is at its most novel and anxiogenic), the half hour prior to the light shock or sound shock stimulus and the hour post light shock or sound shock stimulus were all recorded.

### Drug treatment

For the pharmacological manipulations 1% agar with 5% sucrose was used as a vehicle medium. The tyrosine hydroxylase inhibitor, 3-iodo-L-tyrosine (Sigma-Aldrich, Stockholm, Sweden) was mixed into the medium to a final concentration of 3 mM. There day old male flies were transferred and kept on the media for 48 hours.

### Drosophila brain immunohistochemistry

Male flies were anesthetized and decapitated, and the proboscis was removed. Heads were placed into a staining glass bowl containing 4% paraformaldehyde in 0.1 M sodium phosphate buffer (PBS) and left to fixate in the dark for 2 hr on ice. After fixation, heads were placed in a petri dish containing 4% agarose, and brains were dissected under a light microscope using fine forceps. Brains were washed four times for 15 min each with 0.1 M PBS. Tissues were blocked in 10% normal goat serum (NGS) for 1 hr. The NGS was then discarded, and tissues were incubated with primary antibodies (Millipore, Darmstadt, Germany, anti-GFP chicken polyclonal diluted 1:200 and Immunostar, Wisconsin, USA, mouse anti-Tyrosine hydroxylase 1:500), diluted in 0.01 M PBS containing 0.25% Triton X-100 (PBX) for 2 days at 4°C. Bowls were sealed with parafilm and aluminium foil. Following 48 hr of incubation, brains were washed four times for 15 min each with 0.01 M PBX and incubated with secondary antibody (ThermoFisher Scientific, MA, USA, goat anti-chicken Alexa Fluor 488 and goat anti-mouse Alexa Fluor 594 goat anti-rabbit), diluted 1:1000 in 0.01 M PBX, overnight. Bowls were sealed with parafilm and aluminum foil. Tissues were washed once with 0.01 M PBX for 15 min and twice with 0.01 M PBS for 15 min. Samples were mounted in Vectashield (Vector Laboratories, Peterborough, UK). Images were captured on a Zeiss LSM 510 confocal microscope and visualized using ImageJ.

### In situ hybridization

#### Design and synthesis of RNA probes

Antisense probes were generated from commercial mouse cDNA clones (Source BioScience). The clones were sequenced (Eurofins MWG Operon, Germany) and verified to be correct. Plasmids were linearized with appropriate restriction enzymes and the probes were synthesized using 1μg vector as template with T7, Sp6 or T3 RNA polymerase in the presence of digoxigenin (DIG)-labeled 11-UTP (Roche Diagnostics, Switzerland). Probes were controlled and quantified using the Nanodrop ND-1000 Spectrophotometer (NanoDrop Technologies, Delaware, USA).

#### In situ hybridization on free floating sections

Free floating sections were washed in PBT followed by 6% hydrogen peroxide treatment at room temperature. After successive washing in PBT the sections were treated with 20ug/ml proteinase K (Invitrogen). The sections were post-fixed in 4% formaldehyde before pre-incubation in hybridization buffer (50% formamide, 5xSCC ph4.5, 1% SDS, 50ug/ml tRNA (Sigma Aldrich, Stockholm, Sweden), 50ug/ml heparin (Sigma Aldrich, Stockholm Sweden) in PBT). Hybridization of sections in presence of 100ng probe/ml was performed overnight at 58°C. To remove unbound probe the sections were washed with buffer 1 (50% formamide, 2xSSC ph4.5 and 0.1% Tween-20 in PBT) followed by buffer 2 (50% formamide, 0.2XSSC ph 4.5 and 0.1% Tween-20 in PBT). The sections were incubated in blocking solution (1% blocking reagent (Roche Diagnostics, Sweden, Stockholm) followed by overnight incubation combined with 1:5000 diluted anti-digoxigenin alkaline phosphates conjugated antibody (Roche Diagnostics Scandinavia, Stockholm, Sweden). Unbound antibody were washed away with sequential washes with 0.1% Tween-20 tris-buffered saline (TBST). The sections were then developed with Fast Red (Roche diagnostics, Stockholm, Sweden).

### Ethical statement

All animal procedures were approved by the local ethical committee in Uppsala (permit C 488/12) and follows international guidelines.

### Pairwise linkage disequilibrium

All SNPs in ETV5 or close to ETV5, and that have been associated with BMI and/or dipolar disorder in genome-wide association studies (GWAS) were selected: rs1356292, rs1516725, rs1516726, rs4234589, rs4686728, rs4686730, rs6777779, rs6809651, rs7433760, rs9824882, rs7647305 and rs9816226. P-values of association with BMI and/or bipolar disorder were retrieved from GWAS central [39]. Pairwise linkage disequilibrium (LD) between the SNPs were retrieved from SNAP proxy [83], using “1000 Genome Pilot 1” as the SNP data set, and CEU as the population panel. Fig 9C was generated using the R package LDheatmap [84].

### Statistical analysis

Mean and standard error from all replicates of each experiment was calculated. All analysis was performed with GraphPad Prism 4, and used ANOVA with appropriate post hoc analysis for multiple comparisons (See figure legends for which analysis was performed for each assay).

## Supporting Information

S1 Dataset Excel file containing information about all differentially expressed genes in adult males where Ets96B (*Ets96B*^*RNAi1*^) has been specifically knocked down in the nervous system throughout development. (XLSX)

S1 Fig Schematic diagram of Ets96B genetic region. Exons represented by black boxes, introns represented by a line, the open reading frame (ORF) is represented as a blue box. The enhancer GMR59D04 used to produce the Ets96B-GAL4 transgenic line by Pfeiffer et al (2008) 31 is indicated by a line.
(TIF)

S2 Fig A CAFE assay was used to assess the total food intake in 5–7 days old adult males. (A) Ets96B knocked down throughout development using either the pan-neuronal driver *elav-GAL4* or *Ets96B-GAL4*. Only *Ets96B*^*RNAi1*^ was used for this assay. (n = 50 males per genotype, one-way ANOVA with Tukey’s post hoc test for multiple comparisons.) Error bars = SEM. (B) *Ets96B* knocked down only in adults using the pan-neuronal driver and temperature sensitive allele of the GAL4 inhibitor GAL80 *elav-GAL4*, *tub-Gal80*^*ts*^. Only *Ets96B*^*RNAi1*^ was used for this assay. (n = 50 males per genotype, one-way ANOVA with Tukey’s post hoc test for multiple comparisons.) Error bars = SEM
(TIF)

S3 Fig (A) Relative expression level of *GFP* in 5–7 day old control males (*elav-GAL4;UAS-GFP*) or males where *GFP* was knocked down in the entire nervous system throughout development. This assay was repeated at least 7 times. (n = 25 males per treatment; ** P<0.005 compared with controls, one-way ANOVA with Tukey’s post hoc test for multiple comparisons). Error bars = SEM. (B) Relative expression levels of *CaBP1*, *ERp60* and *Crc* in 5–7 day old control males (*elav-GAL4;UAS-GFP*) or males where *GFP* was knocked down in the entire nervous system throughout development. This assay was repeated at least 7 times. Error bars indicate SEM. (n = 25 males per treatment, one-way ANOVA with Tukey’s post hoc test for multiple comparisons). (C) Triglyceride levels were determined in male control and GFP knockdown flies at 0, 12 and 24 hours of starvation. (n = 30 males per treatment, assay was repeated at least 10 times for each genotype, one-way ANOVA with Tukey’s post hoc test for multiple comparisons). (D) The DAMS system was used to monitor locomotion in control and GFP knockdown males prior to and after light stimulation. (n = 30–60 males per strain; One-way ANOVA with Tukey’s post hoc test for multiple comparisons) In all graphs error bars = SEM.
(TIF)

S4 Fig Genome Browser and Encode Roadmap were used to determine the ETV 5 methylation state in various brain regions. *ETV5* SNPs linked to BMI (red) or BMI and biopolar disorder (blue) are shown at the bottom. Brain Anterior Caudate (BAC), Brain Cingulate Gyrus (BCG), Brain Angular Gyrus (BAG), Brain Hippocampus Middle (BHM), Brain Inferior Temporal Lobe (BITL), Brain Mid Frontal Lobe (BMFL), Brain Substantia Nigra (BSN), Fetal Brain (FB).
(TIF)

S1 Table Table of graded levels of *Etv5* mRNA expression in the mouse brain (PDF)

S2 Table Table containing information about human *ETV5* localized SNPs associated with body mass index (BMI) and/or bipolar disorder. (DOCX)
