# Supplementary figures and images for: The Drosophila ETV5 Homologue Ets96B: Molecular Link between Obesity and Bipolar Disorder
Source: PLoS Genet. 2016 Jun 9;12(6):e1006104. doi: 10.1371/journal.pgen.1006104 (PMC4900636; doi:10.1371/journal.pgen.1006104)

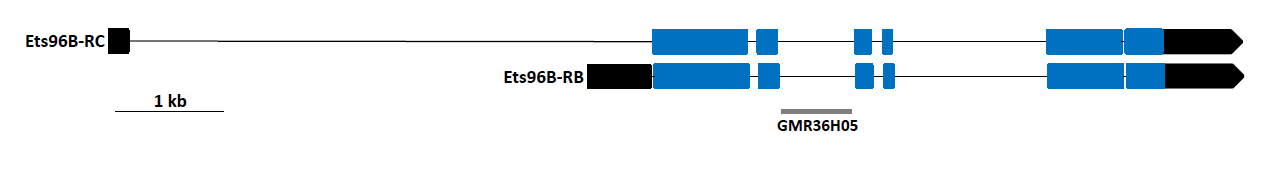

Supplement: S1 Fig — Exons represented by black boxes, introns represented by a line, the open reading frame (ORF) is represented as a blue box. The enhancer GMR59D04 used to produce the Ets96B-GAL4 transgenic line by Pfeiffer et al (2008) 31 is indicated by a line. (TIF) [file pgen.1006104.s002.tif]

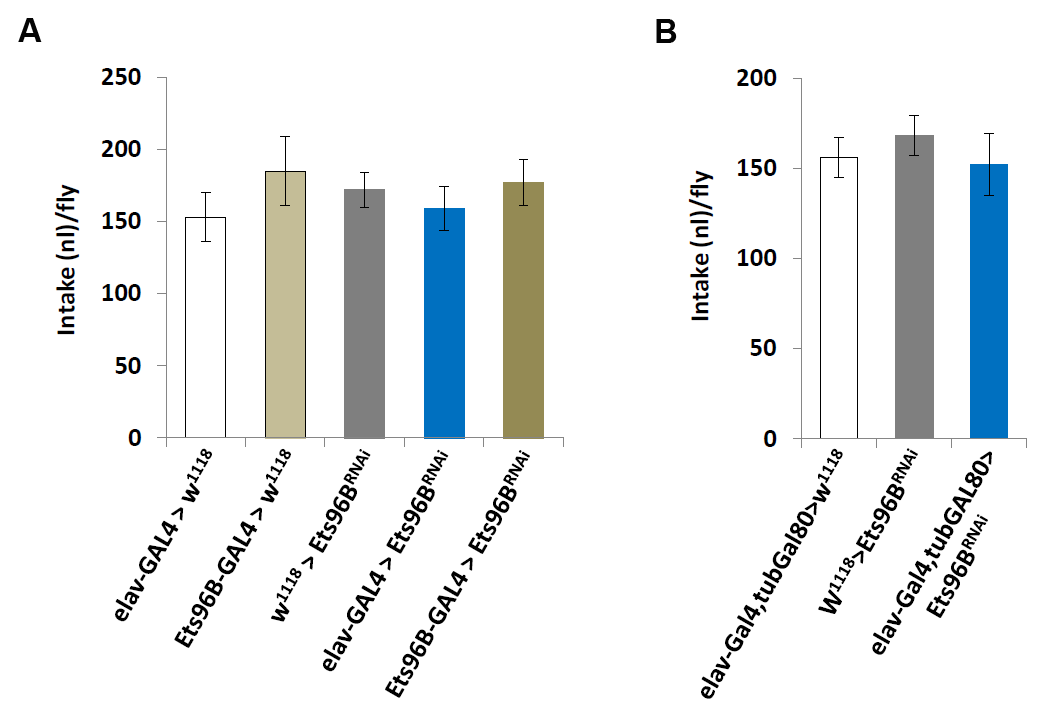

Supplement: S2 Fig — (A) Ets96B knocked down throughout development using either the pan-neuronal driver elav-GAL4 or Ets96B-GAL4. Only Ets96BRNAi1 was used for this assay. (n = 50 males per genotype, one-way ANOVA with Tukey’s post hoc test for multiple comparisons.) Error bars = SEM. (B) Ets96B knocked down only in adults using the pan-neuronal driver and temperature sensitive allele of the GAL4 inhibitor GAL80 elav-GAL4, tub-Gal80ts. Only Ets96BRNAi1 was used for this assay. (n = 50 males per genotype, one-way ANOVA with Tukey’s post hoc test for multiple comparisons.) Error bars = SEM (TIF) [file pgen.1006104.s003.tif]

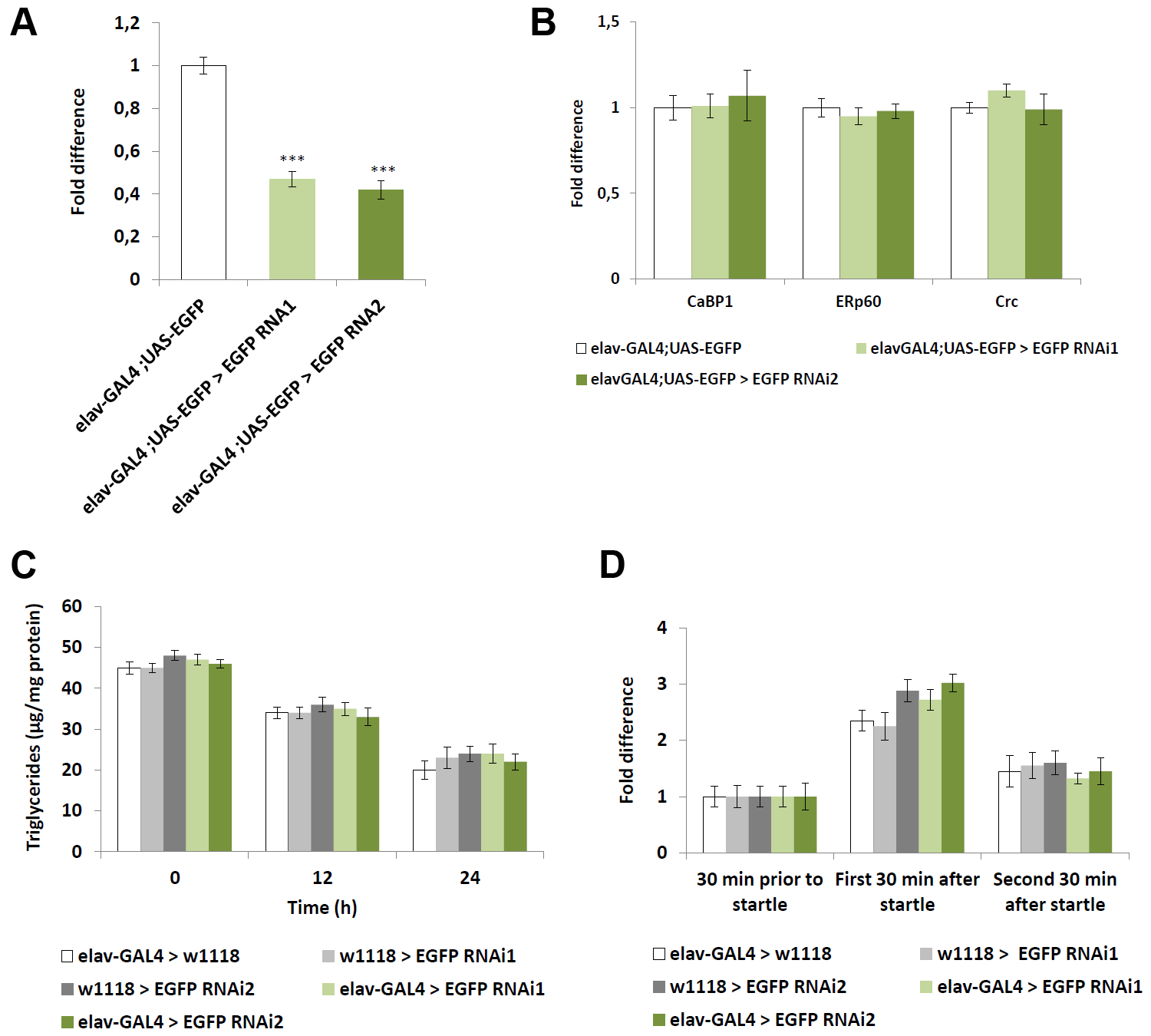

Supplement: S3 Fig — (A) Relative expression level of GFP in 5–7 day old control males (elav-GAL4;UAS-GFP) or males where GFP was knocked down in the entire nervous system throughout development. This assay was repeated at least 7 times. (n = 25 males per treatment; ** P<0.005 compared with controls, one-way ANOVA with Tukey’s post hoc test for multiple comparisons). Error bars = SEM. (B) Relative expression levels of CaBP1, ERp60 and Crc in 5–7 day old control males (elav-GAL4;UAS-GFP) or males where GFP was knocked down in the entire nervous system throughout development. This assay was repeated at least 7 times. Error bars indicate SEM. (n = 25 males per treatment, one-way ANOVA with Tukey’s post hoc test for multiple comparisons). (C) Triglyceride levels were determined in male control and GFP knockdown flies at 0, 12 and 24 hours of starvation. (n = 30 males per treatment, assay was repeated at least 10 times for each genotype, one-way ANOVA with Tukey’s post hoc test for multiple comparisons). (D) The DAMS system was used to monitor locomotion in control and GFP knockdown males prior to and after light stimulation. (n = 30–60 males per strain; One-way ANOVA with Tukey’s post hoc test for multiple comparisons) In all graphs error bars = SEM. (TIF) [file pgen.1006104.s004.tif]

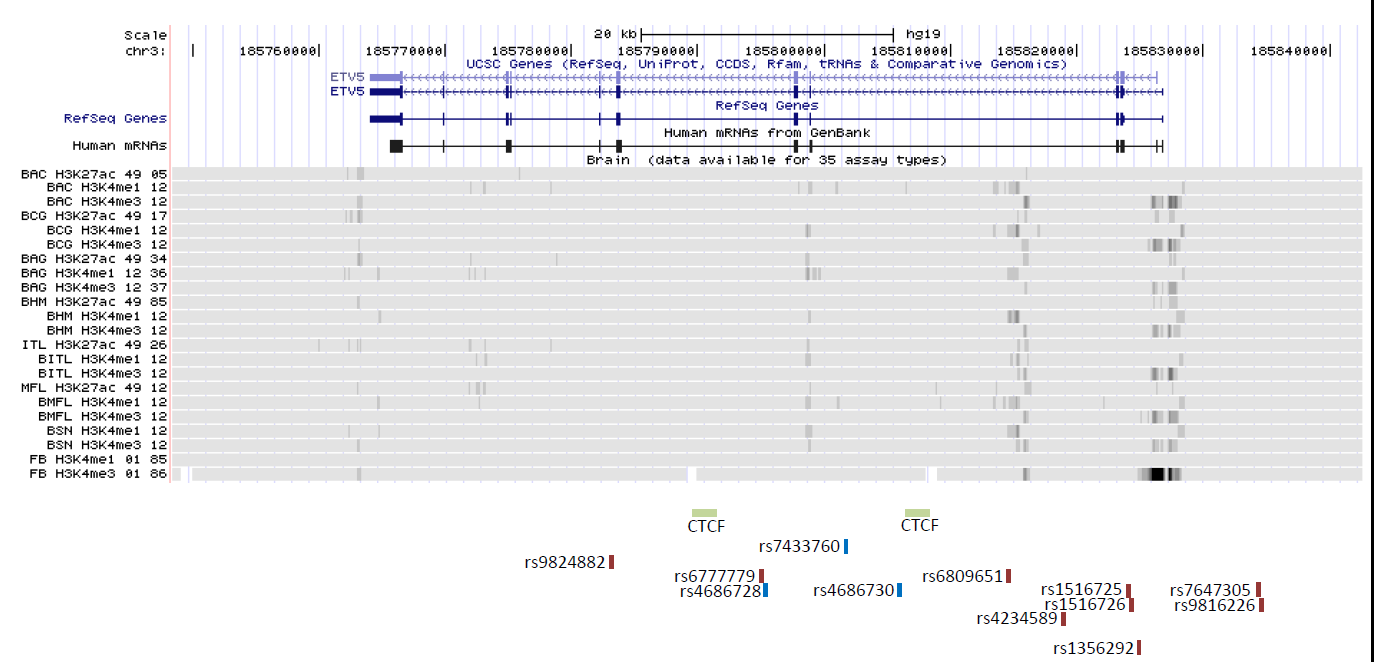

Supplement: S4 Fig — ETV5 SNPs linked to BMI (red) or BMI and biopolar disorder (blue) are shown at the bottom. Brain Anterior Caudate (BAC), Brain Cingulate Gyrus (BCG), Brain Angular Gyrus (BAG), Brain Hippocampus Middle (BHM), Brain Inferior Temporal Lobe (BITL), Brain Mid Frontal Lobe (BMFL), Brain Substantia Nigra (BSN), Fetal Brain (FB). (TIF) [file pgen.1006104.s005.tif]
