# Supplementary material for: The Drosophila ETV5 Homologue Ets96B: Molecular Link between Obesity and Bipolar Disorder
Source: PLoS Genet. 2016 Jun 9;12(6):e1006104. doi: 10.1371/journal.pgen.1006104 (PMC4900636; doi:10.1371/journal.pgen.1006104)
Supplement: S1 Table — (PDF) [file pgen.1006104.s006.pdf]

Supplementary Table 1: Graded levels of Etv5 mRNA expression in the mouse brain

Site	Expression
**Telencephalon**	
Medal septal nuclei (MS)	–
Ventral pallidum	+
**Amygdala**	
Basolateral amygdaloid nu, anterior (BLA)	++
Basolateral amygdaloid nu, ventral (BLV)	++
Basolateral amygdaloid nu, posterior (BLP)	+
Basomedial amygdaloid nu, anterior (BMA)	+
Basomedial amygdaloid nu, posterior (BMP)	+
Central amygdaloid nu, caspular dic (CeC)	+
Central amygdaloid nu, lateral div (CeL)	++
Intercalated nu amygdala (I)	–
Lateral amygdaloid nu, dorsolateral (LaDL)	–
Lateral amygdaloid nu, ventromedial (LaVM)	–
**Cerebral cortex**	
Dorsal endopiriform nu (DEn)	+++
Layer 1 (1)	–
Layer 2 (2)	+
Layer 3 (3)	–
Layer 4 (4)	+++
Layer 5 (5)	++
Layer 6 (6)	+++
Piriform cortex (Pir)	++
Ventral endopiriform nu (VEn)	+++
**Hippocampal formation**	
Granule layer, dentate gyrus (GrDG)	–
Lacunosum molecular layer, hipp (LMol)	–
Molecular layer dentate gyrus (Mol)	–
Oriens layers, hippocampus (Or)	+
Polymorph layer, dentate gyrus (PoDG)	+
Pyramidal cell layers, hippocampus (Py)	+++
Stratum radiatum, hippocampus (Rad)	–
**Diencephalon**	
**Hypothalamus**	
Arcuate hypothalamic nu, dorsal (ArcD)	+
Arcuate hypothalamic nu, lateral (ArcL)	+
Dorsomedial hypothalamic nu (DM)	+
Lateral hypothalamic area (LH)	+
Paraventricular hypothalamic lateral, magnocellular part (PaLM)	+
Paraventricular hypothalamic medial, magnocellular part (PAMM)	+

Site	Expression
Periventricular hypothalamic nu (Pe)	–
Posteror hypthoalamic nu (PH)	–
Ventromedial hypothalamic nu, central (VMHC)	–
**Thalamus**	
Angular thalamic nu (Ang)	–
Anterodorsal thalamic nu (AD)	–
Anteromedial thalamic nu (AM)	–
Anteroventral thalamic nu (AV)	+
Central medial thalamic nu (CM)	–
Dorsal lateral geniculate nu (DLG)	+
Ethmoid thalamic nu (Eth)	–
Interanterior dorsal thalamic nu (AD)	+
Intermediodorsal thalamic nu (IMD)	–
Lat post thalamic nu, laterorostral (LPLR)	+
Lat post thalamic nu, medorostral (LPMR)	+
Lateral habenular nu (LHb)	–
Laterodorsal thalamic nu (LD)	+
Mamillotalamic tract (mt)	–
Medial habenular nu (MHb)	–
Medial geniculate nu, dorsal (MGD)	+
Medial geniculate nu, ventral (MGV)	+
Mediodorsal thalamic nu (MD)	–
Nigrostrital nucleus	++
Oval paracentral nu (OPC)	–
Paracentral thalamic nu (PC)	+
Parafascicular thalamic nu (PF)	–
Parataenial thalamic nu (PT)	–
Paraventricular thalamic nu (PV)	+
Posterior thalamic nu group (Po)	+
Posteromedian thalamic nu (PoMn)	–
Peticular thalamic nu (Rt)	–
Reuniens thalamic nu (Re)	–
Rhomboid thalamic nu (Rh)	–
Stria medullaris, thalamus (STIA)	+++
Submedius thalamic nu (Sub)	+
V posteromed thalamic, nu, parvicel (VPPC)	+
Ventral posterolat thalamic nu (VPL)	+
Ventral posteromed thalamic nu (VPM)	+
Ventral reunions thalamic nu (VRe)+	
Ventrolateral geniculate nu (VLG)	–
Ventrolateral thalamic nu (VL)	+
Ventromed thalamic nu (VM)	+
Zona inserta, dorsal (ZID)	–
Zona inserta, ventral (ZIV)	–

Site	Expression
**Mesencephalon**	
Central nu inferior colliculus (CIC)	+
Dorsal cortex, inferior colliculus (DCIC)	+
Dorsal raphe nu, inferior (DRI)	+
Dorsal raphe, caudal part (DRC)	–
Dorsal raphe nu, ventral (DRV)	–
External cortex, inferior colliculus (ECIC)	+
**Pons**	
Kölliker-Fuse nu (KF)	+
Laterodorsal tegment nu, ventral (LDTgV)	–
Lateral parabrachial nu (LPB)	+
Motor trigmenial nu (Mo5)	+
Ventral tegmental nu (VTg)	+
**Medulla**	
Ambiguus nu (Amb)	+
Area postrema (AP)	–
Dorsal motor nu vagus n (10)	+
Fascial nu (7)	–
Gigantocellular reticular nu, alpha (GiA)	+
Hypoglossal nu (12)	–
Inferior olive, beta subnu (IOBe)	–
Inferior olive, cap of Kooy med nu (IOK)	+
Inferior olive, dorsal accessory nu (IOD)	–
Inferior olive, dorsomed cell grp (IODM)	–
Inferior olive, dorsomed cell col (IODMC)	–
Inferior olive, med nu (IOM)	+
Inferior olive, principal nu (IOPr)	+
Inferior olive, subnu B of med nu (IOB)	–
Inferior olive, subnu C of med nu (IOC)	+
Medial vestibular nu (MVe)	–
nu of solitary tract, commissural (SolC)	–
nu of solitary tract, dorsolateral tract (SolDL)	+
nu of solitary tract, medial (SolM)	–
nu of solitary tract, ventrolateral (SolVL)	–
Prepositus hypoglossal nu (Pr)	+
Pyramidal tract (py)	–
Raphe magnus nu (RMg)	+
Raphe obscurus nu (Rob)	+
Raphe pallidus (RPa)	–
Solitary tract (sol)	+
**Cerebellum**	
Granular layer	+
Molecular layer	–
Purkinje cell layer	+
