# Supplementary material for: The Drosophila ETV5 Homologue Ets96B: Molecular Link between Obesity and Bipolar Disorder
Source: PLoS Genet. 2016 Jun 9;12(6):e1006104. doi: 10.1371/journal.pgen.1006104 (PMC4900636; doi:10.1371/journal.pgen.1006104)
Supplement: S2 Table — (DOCX) [file pgen.1006104.s007.docx]

**Supplementary Table 2: ETV5 localized SNPs associated with body mass index (BMI) and/or bipolar disorder**

| **GWAS Central marker ID** | **Marker accession number** | **BMI**  **p-value** | **Bipolar disorder**  **p-value** | **References** |
| --- | --- | --- | --- | --- |
| HGVM1499284 | rs1356292 | 0.0233 |  | ^1, 2^ |
| HGVM1509502 | rs1516725 | 4e-08 |  | ^2, 3^ |
| HGVM1509503 | rs1516726 | 0.000123 |  | ^1, 2^ |
| HGVM2451169 | rs4234589 | 2.17e-11 |  | ^1, 2^ |
| HGVM2684660 | rs4686728 | 5.2e-08 | 0.02149 | ^1, 2, 4^ |
| HGVM2684662 | rs4686730 | 5.5e-08 | 0.01012 | ^1, 2, 4^ |
| HGVM7105524 | rs6777779 | 0.0357388 |  | ^1, 2^ |
| HGVM7135952 | rs6809651 | 4.32e-11 |  | ^1, 2^ |
| HGVM7707562 | rs7433760 | 0.000631 | 0.03211 | ^1, 2, 4^ |
| HGVM8815802 | rs9824882 | 4.85e-08 |  | ^1, 2^ |

1. Speliotes EK*, et al.* Association analyses of 249,796 individuals reveal 18 new loci associated with body mass index. *Nature genetics* **42**, 937-948 (2010).

2. Hindorff LA*, et al.* Potential etiologic and functional implications of genome-wide association loci for human diseases and traits. *Proc Natl Acad Sci U S A* **106**, 9362-9367 (2009).

3. Berndt SI*, et al.* Genome-wide meta-analysis identifies 11 new loci for anthropometric traits and provides insights into genetic architecture. *Nat Genet* **45**, 501-512 (2013).

4. Smith EN*, et al.* Genome-wide association study of bipolar disorder in European American and African American individuals. *Mol Psychiatry* **14**, 755-763 (2009).
